# Development and validation of a predictive model combining clinical, radiomics, and deep transfer learning features for lymph node metastasis in early gastric cancer

**DOI:** 10.3389/fmed.2022.986437

**Published:** 2022-10-03

**Authors:** Qingwen Zeng, Hong Li, Yanyan Zhu, Zongfeng Feng, Xufeng Shu, Ahao Wu, Lianghua Luo, Yi Cao, Yi Tu, Jianbo Xiong, Fuqing Zhou, Zhengrong Li

**Affiliations:** ^1^Department of Gastrointestinal Surgery, The First Affiliated Hospital, Nanchang University, Nanchang, Jiangxi, China; ^2^Institute of Digestive Surgery, The First Affiliated Hospital of Nanchang University, Nanchang, Jiangxi, China; ^3^Medical Innovation Center, The First Affiliated Hospital of Nanchang University, Nanchang, China; ^4^Department of Radiology, The Second Affiliated Hospital of Soochow University, Suzhou, Jiangsu, China; ^5^Department of Radiology, The First Affiliated Hospital, Nanchang University, Nanchang, Jiangxi, China; ^6^Department of Pathology, The First Affiliated Hospital of Nanchang University, Nanchang, Jiangxi, China

**Keywords:** early gastric cancer (EGC), lymph node metastasis, deep learning, radiomics, convolutional neural networks

## Abstract

**Background:**

This study aims to develop and validate a predictive model combining deep transfer learning, radiomics, and clinical features for lymph node metastasis (LNM) in early gastric cancer (EGC).

**Materials and methods:**

This study retrospectively collected 555 patients with EGC, and randomly divided them into two cohorts with a ratio of 7:3 (training cohort, *n* = 388; internal validation cohort, *n* = 167). A total of 79 patients with EGC collected from the Second Affiliated Hospital of Soochow University were used as external validation cohort. Pre-trained deep learning networks were used to extract deep transfer learning (DTL) features, and radiomics features were extracted based on hand-crafted features. We employed the Spearman rank correlation test and least absolute shrinkage and selection operator regression for feature selection from the combined features of clinical, radiomics, and DTL features, and then, machine learning classification models including support vector machine, K-nearest neighbor, random decision forests (RF), and XGBoost were trained, and their performance by determining the area under the curve (AUC) were compared.

**Results:**

We constructed eight pre-trained transfer learning networks and extracted DTL features, respectively. The results showed that 1,048 DTL features extracted based on the pre-trained Resnet152 network combined in the predictive model had the best performance in discriminating the LNM status of EGC, with an AUC of 0.901 (95% CI: 0.847–0.956) and 0.915 (95% CI: 0.850–0.981) in the internal validation and external validation cohorts, respectively.

**Conclusion:**

We first utilized comprehensive multidimensional data based on deep transfer learning, radiomics, and clinical features with a good predictive ability for discriminating the LNM status in EGC, which could provide favorable information when choosing therapy options for individuals with EGC.

## Background

Gastric cancer (GC) is one of the most commonly diagnosed malignant tumors and the third leading cause of cancer-related deaths in China (1, 2). Early gastric cancer (EGC) is defined as a lesion of the stomach that invades no more than the submucosa, regardless of lymph node metastasis (LNM) (3). With the popularization of endoscopic technology, patients with EGC can be more easily diagnosed, accounting for approximately 20% of GC in China (4). The 5-year overall survival rate in EGC was greater than 90% after standardized D1 lymphadenectomy treatment. Regional node metastasis is an important prognostic factor for patients with EGC. However, only between 0 and 20% of patients who had a radical gastrectomy developed LNM, and the majority of these individuals had received excessive surgical treatment (5, 6). According to the Chinese recommendations for diagnosing and treating gastric cancer and the Japanese Gastric Cancer Association treatment guidelines, endoscopic submucosal dissection (ESD) is authorized as a curative therapy option for patients with EGC with a low risk of LNM (7, 8). Due to its minimally invasive, function preservation, and better postoperative quality of life, ESD has become a more acceptable therapeutic method than surgical procedures in treating EGC recently (9, 10). As a result, knowing the status of LNM is critical when choosing therapy options for individuals with EGC.

Recently, many efforts had been explored to identify clinical or pathological biomarkers to predict the LNM of gastric cancer. For example, several research studies had found independent high-risk factors for EGC lymph node metastases and developed prediction models, such as clinical features, genetic characteristics, and imaging data (4, 11, 12). For clinical features, high-risk indicators included age, sex, ulceration, invasion depth, histological types, differentiation, tumor size, serum indices, and lymphovascular invasion in several prediction models (11, 13, 14). Daisuke and colleagues (15) established a reliable diagnostic tool based on a 15-gene signature to predict LNM in patients with EGC. The HER2 status can also improve accuracy of predicting LNM (12). However, these aforementioned high-risk factors for predicting the LNM status in EGC could not effectively reduce excessive surgical treatment with standard D1 lymph node dissection. In the meantime, previous research studies almost used mono-modal data containing limited information to develop a model to assess the possibility of LNM, making it difficult to improve its accuracy. Therefore, accurate prediction of the LNM status of EGC has become a bottleneck stage.

Non-invasive computed tomography (CT) is proposed as the first-line imaging tool for identifying LNM by the National Comprehensive Cancer Network, which is frequently utilized in patients with gastric cancer for differential diagnosis and preoperative diagnosis, treatment evaluation, and staging, and this technology can facilitate the detection of malignant lesions (16, 17). Jingtao et al. (4) demonstrated that the sum of long-diameter and the sum of short-diameter lymph nodes greater than 3 mm in CT images were available indicators to diagnose LNM in EGC. However, the accuracy of CT discriminating the LNM status is only approximately 60% and even lower in ECG (13), which is an unsatisfactory clinical level of diagnosis.

Radiomics refers to the conversion of medical images into high-dimensional quantitative data that can be used to characterize microscopic aspects of malignant tissues (18). Deep convolutional neural networks (CNNs) have achieved significant results in recent years in the field of computer vision, which serves a similar function in medical imaging (16, 17, 19). In medical imaging, the successful implementation of the aforementioned methods necessitates a sufficient number of the training cohort. However, acquiring a large number of medical images is difficult (20). Due to a pre-trained CNN known as “transfer learning (TL)” can be used to minimize overfitting with a small training size, TL has gradually been used in various medical image analysis domains in recent years (21, 22). TL increases model performance in target tasks by transferring previously learned features from source tasks. A previous study found that a TL radiomics nomogram based on gastric whole slide images can assist in distinguishing primary gastric lymphoma from Borrmann type IV GC (22). In addition, Linlin et al. (21) developed a convenient model based on deep learning-based radiomics characteristics to differentiate brain abscess from cystic glioma. Therefore, it suggests that building a TL radiomics model may be beneficial in improving the accuracy of LNM prediction in EGC.

Currently, only a few type of research focused on evaluating the efficacy of deep learning-based radiomics for LNM prediction in GC, and research mostly has concentrated on advanced GC (23, 24), while yet to be reported in EGC. Therefore, this study aimed to create a predictive model for discriminating the LNM status in EGC, combining clinical indicators, radiomics features, and pre-trained CNN-identified deep learning features.

## Materials and methods

### Patients

**FIGURE 1:**
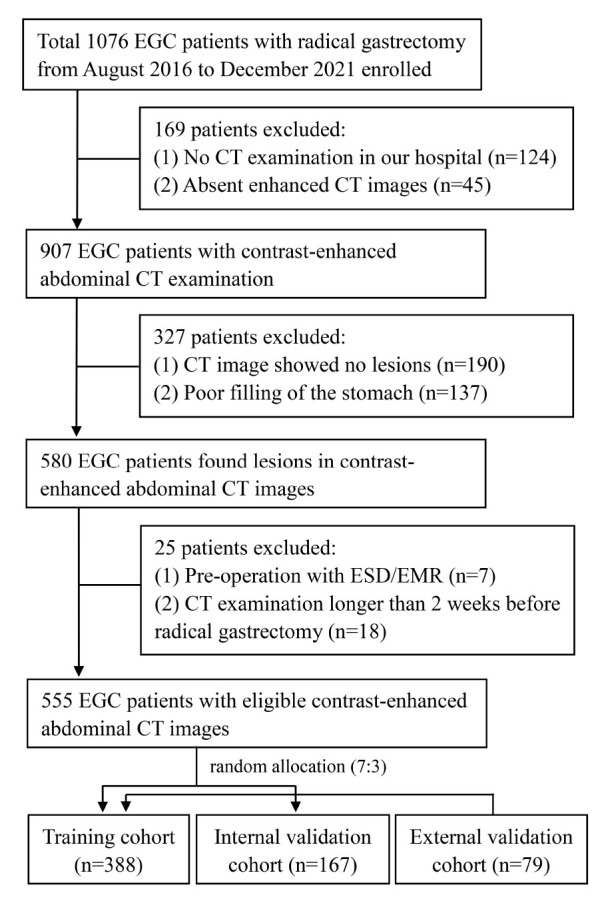
Inclusion and exclusion criteria for patients with EGC for the training and internal validation cohorts. EGC, early gastric cancer; CT, computed tomography; ESD, endoscopic submucosal dissection.

The Ethics Committee of the First Affiliated Hospital of Nanchang University approved this retrospective study and waived the necessity for informed consent. Between August 2016 and December 2021, we collected patients with EGC who had a radical gastrectomy at the First Affiliated Hospital of Nanchang University. Overall, of 1,076 patients with EGC, 555 patients who had radical gastrectomy satisfied the following criteria (Figure 1). Eligible patients were those who had a radical gastrectomy with standard D1/D2 lymph node dissection and had pathologically proven EGC and were treated for the first time. In total, 79 patients with EGC collected from the Second Affiliated Hospital of Soochow University were regarded as the external validation cohort. The exclusion criteria were as follows: (1) no preoperative CT imaging available, (2) patients with low CT imaging quality cannot be used to further analyze, (3) patients with ESD or other therapy before surgery, (4) patients with insufficient clinical information, and (5) CT scanned more than 2 weeks before surgery. The patients were randomly split into two cohorts, with a ratio of 7:3—the training cohort (*n* = 388) and the internal validation cohort (*n* = 167).

### Clinical characteristics

The clinical features of the patients with EGC we collected included age, gender, tumor size, depth of tumor infiltration, histological grade, Lauren type, ulcer, and lymphovascular invasion (Supplementary Datasheet 1). Tumor size was determined as the maximal diameter, and depth was measured at the deepest point of infiltrated carcinoma cells.

### Computed tomography scanning protocol

128-channel CT (Siemens Healthcare), 256-channel CT (Siemens Healthcare), 128-channel CT (IQon Spectral CT), and 256-channel CT (Philips Brilliance iCT 256) were used for contrast-enhanced CT scanning. The scanning parameters were a tube voltage of 80 to 120°kVp, a tube current of 120–300°mAs, a pitch of 0.6 to 1.25 mm, an image matrix of 512 × 512, and a reconstruction slice thickness of 1 or 2 mm. All patients received racanisodamine hydrochloride injection of 20 mg by intramuscular injection and drank 1,000–2,000 mL of water before abdomen contrast-enhanced CT. The arterial phase and portal venous phase were obtained within 25–30 s and 65–70 s, respectively, following intravenous administration of contrast media (1.5°mL/kg, at a rate of 3.0–3.5 ml/s).

### Image preprocessing and tumor segmentation

In this study, we used ITK-SNAP software (version 3.6.0, USA) to manually segment regions of interest (ROIs). The tumor lesion was clearly enhanced and more readily distinguished between the tumor and peripheral normal tissue during the portal venous phase, and many prior investigations used this phase to segment tumor lesions (25, 26). The lesion was considered visible and employed for the following segmentation when the characteristic of the lesion on the CT images was consistent with the pathological results. We meticulously outlined neighboring upper and lower slices of the solid tumor in the three-dimensional (3D) medical imaging, being cautious not to include the normal stomach wall or surrounding air or fluid. Then, a radiologist (YZ, 4 years of experience) segmented all 634 patients with EGC. The intra-/inter-class correlation coefficient (ICC) was used to evaluate the reproducibility of the radiomics feature (27). To keep the repetitive and stable radiomics parameters, we selected 30 patients, and then, the ROIs (YZ) were redrawn a month later for feature extraction. The ROIs of these 30 patients were outlined by another radiologist (FZ, with 12 years of experience) to ensure interobserver repeatability.

**FIGURE 2:**
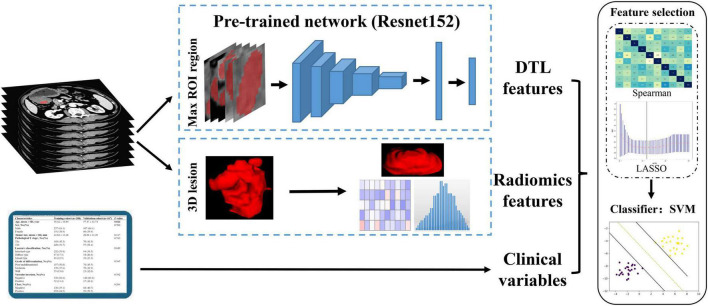
Workflow of model development. DTL, deep transfer learning; ROI, regions of interest.

Since the deep transfer learning (DTL) model input was rectangular images comprising the full ROI lesions, the maximal sliced photo of the tumor lesion for each patient was chosen as the model input (23). The CT image was cropped using a rectangular ROI around the tumor contour. Then, the slices of the rectangular frame were saved in a “png” format for subsequent analysis (Figure 2).

### Feature extraction

A total of 107 radiomics features were traditionally extracted based on 3D ROIs, which are divided into three categories: 14 shape features, 18 first-order statistics features, and 75 texture features (Supplementary Datasheet 2). These feature extractions were performed by using PyRadiomics software (version 2.1.0).

In this study, we represented a TL learning network for overcoming the overfitting problems that regular deep learning suffers from due to insufficient training data. The parameters of several deep learning networks were trained by maximal rectangular slice ROIs of EGC, including Resnet152, Resnet101, Resnet50, Resnet34, Resnet18, Wide_resnet101_2, Wide_resnet50_2, and Inception v3. Then, convolution neural networks based on pre-trained TL networks were used to extract DTL features, which followed the following steps: the slices of ROIs were fed to the pre-trained network; the average probability from all slices was used to generate TL features; and the penultimate FC layer output was used as TL features (21). Based on these pre-trained deep learning networks, we extracted 512–2,048 transfer learning features, respectively (Supplementary Table 6). Furthermore, our research was implemented in Python 3.10 and run on a system with an Intel Xeon Silver 4214 CPU and 256 GB memory.

### Feature fusion

To improve the accuracy of LNM prediction in EGC, we fused clinical variables, radiomics features, and DTL features. The fusion scheme is to combine various features for subsequent analysis. The groups of feature fusion included clinical variables combined with radiomics features, clinical variables combined with DTL features, DTL features combined with radiomics features, and clinical variables combined with radiomics features and DTL features. In addition, we also used mono-modal data to build machine learning classification models.

### Feature selection and model construction

The radiomics parameters’ repeatability and stability were assessed using intraclass correlation coefficients (ICCs). Only radiomics features with an ICC ≥ 0.75 were considered highly stable and retained for subsequent analysis. After feature fusion, we adopted a three-step feature selection method to select the best features for discriminating the LNM status in EGC. First, each feature group separately was used to standardize combined features by z score normalization in the training and validation cohorts. Then, we employed the Spearman rank correlation test to evaluate the linear correlation between individual features for redundancy elimination (28). Once two features have a stronger correlation, they will have a higher absolute value of the correlation coefficient. We selected one of the features for subsequent analysis when a Spearman correlation coefficient > 0.9 between each feature. Finally, the least absolute shrinkage and selection operator (LASSO) regression was utilized for feature selection with non-zero coefficients as valuable predictors in each feature group (29).

**FIGURE 3:**
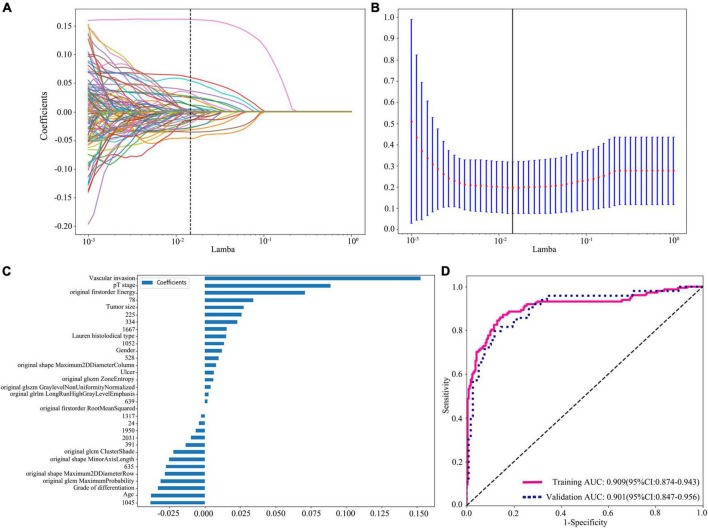
Radiomics + DTL (Resnet152) + clinical features dimension reduction and performance of the model. **(A)** LASSO coefficient profiles of the features. Different color line shows corresponding coefficient of each feature. **(B)** Tuning parameter (λ) selection in LASSO model. **(C)** Selected features weight coefficients. **(D)** Area under the curve (AUC) of predictive model based on radiomics + DTL (Resnet152) + clinical features in training and validation cohorts. DTL, deep transfer learning; LASSO, least absolute shrinkage and selection operator.

After feature selection and fusion, we employed Python Scikit-learn to develop machine learning classification models in each feature group. The machine learning classification models, including support vector machine (SVM), K-nearest neighbor (KNN), random decision forests (RF), and XGBoost, were compared for their different performances. Receiver operating characteristic (ROC) curves and AUC values were used to assess the discriminative ability of the model. Quantitative indicators included accuracy, sensitivity, and specificity (Figure 3).

### Statistical analysis

Chi-square tests or Fisher tests were used to compare categorical variables, while *t*-tests or the Mann–Whitney *U*-test was used to compare quantitative variables to evaluate the differences in patient characteristics. We employed MedCalc software (version 20.100) to calculate differences among different models using the Delong test. Statistical significance was defined as a *P*-value less than 0.05 in a two-sided analysis. We employed IBM SPSS Statistics (Version 20.0, USA) to assess the clinical variables. ICCs, Spearman rank correlation test, z score normalization, and LASSO regression analysis were performed with Python (version 3.10^1^) and R software (version 3.3.1, Austria^2^).

## Results

### Patients characteristics

Table 1 represents the characteristics of all patients. In this study, the training, internal validation, and external validation cohorts included 388, 167, and 79 patients with EGC, respectively. We collected 168 patients with invasion of the mucosa (T1a) and 220 patients with invasion of the submucosa (T1b) in the training cohort, while the internal validation cohort enrolled 70 patients with invasion of the mucosa and 97 patients with invasion of the submucosa. There were 23 patients with invasion of the mucosa and 56 with invasion of the submucosa in the external validation. In these three cohorts, the rates of LNM were 38.14% (148/388), 29.34% (49/167), and 21.52% (17/79) in training, internal validation, and external validation cohorts, respectively. Only the grade of differentiation of EGC in the three cohorts showed significant differences (*P*-value = 0.036). However, the rest of the clinical characteristics including age, gender, tumor size, depth of tumor infiltration, Lauren type, ulcer, and lymphovascular invasion were not significantly different between the training cohort and two validation cohorts.

**TABLE 1:** Characteristics of early gastric cancer (EGC) patient included for classification modeling.

Characteristics	Training cohort (*n* = 388)	Internal validation cohort (*n* = 167)	External validation cohort (*n* = 79)	*P*-value
**Age, mean ± SD, year**	58.62 ± 10.84	57.47 ± 11.74	58.09 ± 12.07	0.535
**Gender, No.(%)**				
Male	237 (61.1)	107 (64.1)	48 (60.8)	0.784
Female	151 (38.9)	60 (35.9)	31 (39.2)	
**Tumor size, mean ± Std, mm**	22.82 ± 13.28	20.88 ± 11.49	20.96 ± 12.09	0.181
**Pathological T stage** ^†^ **, No.(%)**				0.064
T1a	168 (43.3)	70 (41.9)	23 (29.1)	
T1b	220 (56.7)	97 (58.1)	56 (70.9)	
**Lauren’s classification, No.(%)**				0.075
Intestinal type	232 (59.8)	94 (56.3)	46 (58.2)	
Diffuse type	67 (17.3)	34 (20.4)	23 (29.1)	
Mixed type	89 (22.9)	39 (23.3)	10 (12.7)	
**Grade of differentiation, No.(%)**				**0.036**
Poor/undifferentiated	197 (50.8)	76 (45.5)	28 (35.4)	
Moderate	138 (35.6)	70 (41.9)	43 (54.4)	
Well	53 (13.6)	21 (12.6)	8 (10.2)	
**Vascular invasion, No.(%)**				0.608
Negative	336 (86.6)	140 (83.8)	66 (83.5)	
Positive	52 (13.4)	27 (16.2)	13 (16.5)	
**Ulcer, No.(%)**				0.300
Negative	136 (35.1)	68 (40.7)	54 (68.4)	
Positive	252 (64.9)	99 (59.3)	25 (31.6)	

Quantitative variables were in mean ± SD and qualitative variables are in n (%). ^†^According to the eighth edition AJCC Cancer Staging Manual. The bolded *P*-value showed statistically significant (*P*-value < 0.05).

### Results of the feature extraction and selection

A total of 107 radiomics features were traditionally extracted based on three-dimensional ROIs. Only radiomics features with an ICC ≥ 0.75 were considered highly stable and retained for subsequent analysis, and then, we selected 101 radiomics features for the following work, instead of original gldm small dependence low gray level emphasis, original glrlm short run low gray level emphasis, original gldm low gray level emphasis, original glrlm low gray level run emphasis, original glszm small area low gray level emphasis, and original glszm low gray level zone emphasis (Supplementary Table 1). The tumor patch images were fed into the pre-trained CNN, which extracted 512–2,048 DTL features from each CT image modality. The extracted DTL features were output from the pre-trained CNN final fully connected layer, and the pre-trained CNN included Resnet152, Resnet101, Resnet50, Resnet34, Resnet18, Wide_resnet101_2, Wide_resnet50_2, and Inception v3.

All groups of feature fusion were analyzed by the Spearman rank correlation test and LASSO regression, and all features with non-zero coefficients were selected to construct classification models. The final selected features of clinical variables combined radiomics feature group, clinical variables combined DTL feature group, DTL feature combined radiomics feature group, and clinical variable combined radiomics features with DTL feature group are listed in Supplementary Tables 2, 3.

### Performance comparison between various deep transfer learning networks

To find the best model for the LNM status in EGC, we compared the performance of pre-trained Resnet152, Resnet101, Resnet50, Resnet34, Resnet18, Wide_resnet101_2, Wide_resnet50_2, and Inception v3 (Table 2). Various DTL features combining clinical variables and radiomics features were used to construct a diagnostic model. The results showed that pre-trained Resnet152 was the best performance to distinguish the LNM status in EGC with AUC 0.901 (95% CI: 0.847–0.956) and 0.915 (95% CI: 0.850–0.981) in the internal validation and external validation cohorts, respectively. In addition, the internal validation cohort had an accuracy of 96.2%, a sensitivity of 80.0%, and a specificity of 88.1%; meanwhile, the external validation cohort had an accuracy of 86.1%, a sensitivity of 88.2%, and a specificity of 80.6%. In the internal and external validation cohort, the AUC score and accuracy of the Resnet152 model were the best in terms of performance compared to other models, and the validation cohort had the most suitable data to evaluate the generalization ability of the model.

**TABLE 2:** Difference of various deep transfer learning models.

Models	Cohorts	AUC (95% CI)	Accuracy	Sensitivity	Specificity
Resnet152	Training	0.909 (0.874–0.943)	0.830	0.872	0.846
	Internal validation	0.901 (0.847–0.956)	0.962	0.800	0.881
	External validation	0.915 (0.850–0.981)	0.861	0.882	0.806
Resnet101	Training	0.923 (0.889–0.958)	0.835	0.885	0.904
	Internal validation	0.887 (0.829–0.945)	0.826	0.735	0.898
	External validation	0.899 (0.803–0.996)	0.848	0.882	0.839
Resnet50	Training	0.937 (0.909–0.966)	0.869	0.892	0.892
	Internal validation	0.882 (0.825–0.940)	0.850	0.735	0.898
	External validation	0.900 (0.821–0.980)	0.823	0.941	0.790
Resnet34	Training	0.916 (0.883–0.949)	0.832	0.872	0.858
	Internal validation	0.877 (0.817–0.937)	0.832	0.755	0.864
	External validation	0.884 (0.805–0.963)	0.823	0.941	0.694
Resnet18	Training	0.939 (0.911–0.967)	0.838	0.919	0.879
	Internal validation	0.831 (0.761–0.901)	0.820	0.592	0.932
	External validation	0.862 (0.762–0.963)	0.810	0.824	0.806
Wide_resnet101_2	Training	0.921 (0.889–0.954)	0.835	0.865	0.892
	Internal validation	0.859 (0.795–0.922)	0.826	0.755	0.881
	External validation	0.846 (0.742–0.951)	0.747	0.882	0.661
Wide_resnet50_2	Training	0.937 (0.909–0.964)	0.840	0.851	0.896
	Internal validation	0.868 (0.806–0.929)	0.844	0.714	0.907
	External validation	0.888 (0.800–0.976)	0.861	0.706	0.903
Inception v3	Training	0.890 (0.852–0.929)	0.830	0.851	0.846
	Internal validation	0.897 (0.844–0.950)	0.826	0.837	0.839
	External validation	0.900 (0.825–0.976)	0.823	0.765	0.887

AUC, area under the receiver operating characteristic curve; 95% CI, 95% confidence interval.

### Performance comparison between various feature fusions

**FIGURE 4:**
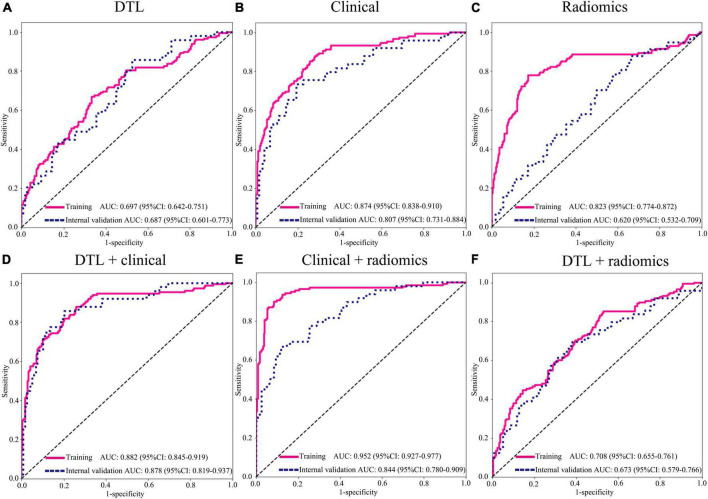
Area under the curve (AUC) of various groups of feature fusion in the training and internal validation cohorts. **(A)** DTL features (Resnet152); **(B)** clinical features; **(C)** radiomics features; **(D)** DTL features (Resnet152) + clinical features; **(E)** clinical + radiomics features; **(F)** DTL features (Resnet152) + radiomics features. DTL, deep transfer learning.

In this study, we compared the modeling effects of the combined modality, including clinical variables combined radiomics feature group, clinical variables combined DTL feature group, and DTL features combined radiomics feature group; meanwhile, three mono-modal features were also used to construct a model to diagnose the LNM status in EGC, respectively (Figure 4, Table 3, and Supplementary Figure 1). The results demonstrated that the predictive model just based on clinical variables with AUC 0.807 (95% CI: 0.731–0.910) had better performance than DTL features with 0.687 (95% CI: 0.600–0.773) and radiomics features with 0.631 (95% CI: 0.540–0.724) in the internal validation cohort, as well as the external validation. Especially, we found that a predictive model based on DTL or radiomics features combined with clinical variables can significantly improve the ability to discriminate the LNM status in EGC with AUCs of 0.878 (95% CI: 0.819–0.937) and 0.844 (95% CI: 0.780–0.910) in the internal validation cohort, and AUCs of 0.913 (95% CI: 0.842–0.986) and 0.849 (95% CI: 0.739–0.959) in the external validation cohort. However, the best modeling performance of the combined modality feature was clinical variables combined with radiomics features with DTL features, and the AUCs were 0.901 (95% CI: 0.847–0.956) and 0.915 (95% CI: 0.850–0.981) in the internal validation and external validation cohorts. In addition, we used the Delong test to compare the different performance between the various prediction models. Supplementary Table 4 shows *P*-values between different models in the two validation cohorts, respectively.

**TABLE 3:** Performance of various combined models.

Models	Cohorts	AUC (95% CI)	Accuracy	Sensitivity	Specificity
DTL features	Training	0.697 (0.642–0.751)	0.660	0.669	0.667
	Internal validation	0.687 (0.601–0.773)	0.725	0.857	0.476
	External validation	0.600 (0.449–0.750)	0.785	0.710	0.607
Clinical variables	Training	0.874 (0.838–0.910)	0.794	0.878	0.717
	Internal validation	0.807 (0.731–0.884)	0.796	0.735	0.805
	External validation	0.882 (0.806–0.959)	0.785	0.941	0.726
Radiomics features	Training	0.823 (0.774–0.872)	0.724	0.770	0.788
	Internal validation	0.620 (0.532–0.709)	0.689	0.776	0.530
	External validation	0.637 (0.464–0.811)	0.747	0.706	0.629
DTL features + clinical variables	Training	0.882 (0.845–0.919)	0.789	0.878	0.742
	Internal validation	0.878 (0.819–0.937)	0.814	0.857	0.797
	External validation	0.913 (0.842–0.986)	0.886	0.765	0.902
Radiomics features + clinical variables	Training	0.952 (0.927–0.977)	0.822	0.900	0.915
	Internal validation	0.844 (0.780–0.909)	0.808	0.673	0.873
	External validation	0.849 (0.739–0.959)	0.835	0.765	0.839
DTL + radiomics features	Training	0.707 (0.655–0.761)	0.660	0.851	0.471
	Internal validation	0.673 (0.579–0.766)	0.719	0.694	0.619
	External validation	0.581 (0.415–0.746)	0.759	0.706	0.541
DTL + radiomics features + clinical variables	Training	0.909 (0.874–0.943)	0.830	0.872	0.846
	Internal validation	0.901 (0.847–0.956)	0.962	0.800	0.881
	External validation	0.915 (0.850–0.981)	0.861	0.882	0.806

AUC, area under the receiver operating characteristic curve; 95% CI, 95% confidence interval.

### Performance comparison among support vector machine, K-nearest neighbor, random decision forests, and XGBoost classification

**FIGURE 5:**
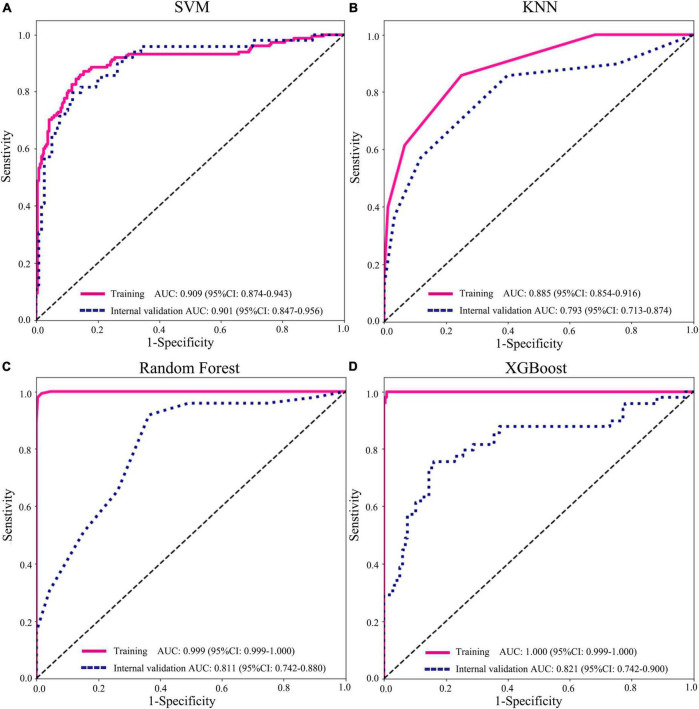
Performance of different machine learning classifications based on radiomics + deep transfer learning (Resnet152) + clinical features in the training and internal validation cohorts. **(A)** support vector machine (SVM); **(B)** K-nearest neighbor (KNN); **(C)** random decision forests; and **(D)** XGBoost.

To find a suitable classifier to develop a diagnostic model, we compared the performance of different machine learning classifications. In the internal and external validation cohorts, the results represented that AUCs of SVM classification were significantly better than those of KNN, RF, and XGBoost classification in various prediction models. For example, in clinical variables combining radiomics features with the DTL feature model, the AUCs of SVM, KNN, RF, and XGBoost were 0.901 (95% CI: 0.847–0.956), 0.793 (95% CI: 0.712–0.874), 0.811 (95% CI: 0.742–0.880), and 0.820 (95% CI: 0.742–0.900) in internal validation (Figure 5 and Supplementary Figure 2). In addition, the accuracy of SVM classification was also better in terms of performance than that in KNN, RF, and XGBoost classification, with accuracy values of 0.962, 0.790, 0.748, and 0.808 in the internal validation and accuracy of 0.861, 0.823, 0.772, and 0.873 in the external validation cohort (Supplementary Table 5).

## Discussion

Currently, the primary method to cure patients with EGC was gastrectomy with D1 lymphadenectomy or endoscopic surgery. With the development of fewer invasion treatments, ESD and EMR were considered curative treatment methods for ECG patients without LNM (8). Furthermore, LNM has been approved as one of the most important prognostic factors, regardless of EGC and advanced GC (30, 31). Therefore, assessing the likelihood of LNM is critical to determining therapy options for patients with EGC. In this study, we developed and validated clinical variables combining radiomics features with the DTL features model to discriminate the LNM status in EGC, which was significantly better than any single model. Especially, this is the first study to combine DTL features to predict the LNM status in EGC.

Previous studies had constructed various models to predict the LNM status in EGC since it was the most important indicator for therapy options and prognosis. Several studies showed that clinicopathological risk factors for LNM in EGC included age, gender, tumor size, depth of invasion, histological type, ulceration, and lymphovascular invasion (11, 32, 33). In our results, based on LASSO accordant regression coefficients, these clinical features were also verified as risk factors, especially for lymphovascular invasion, depth of invasion, and tumor size as the three most important indicators. In the three prediction models, respectively, based on mono-modal data of clinical variables, radiomics, and DTL features, clinical variables represented a better ability to discriminate LNM than the other two single models with AUCs of 0.807 (95% CI: 0.731–0.884) and 0.882 (95% CI: 0.806–0.959) in the internal and external validation cohorts, respectively. According to the latest guidelines for endoscopic submucosal dissection and endoscopic mucosal resection for early gastric cancer (second edition), absolute indications for ESD or EMR mainly depend on clinicopathological risk factors (8). The indications of endoscopic surgery that was approved as a curative treatment method just based on clinicopathological risk factors were only suitable for limited patients with EGC, which may lead to a large number of patients acquiring overtreatment with D1 lymphadenectomy. Some researchers have also paid attention to other related predictors with LNM in EGC, such as hereditary features, visualized features of computed tomography, and endoscopic ultrasonography (4, 12, 33–35). On the EGC CT images among 130 patients, the number, and sum of long diameter and the sum of short diameter of lymph nodes larger than 3 mm showed a better performance to discriminate the LNM status with an AUC greater than 0.75 (4). The deep learning radiomics model constructed by Dong et al. for the prediction of the LNM status in advanced GC showed good discrimination with AUCs of 0.797 (95% CI: 0.771–0.823) and 0.822 (95% CI: 0.756–0.887) in the primary cohort and international validation cohort, respectively. Thus, it is necessary to excavate deeply detailed information on tumor heterogeneity of CT images to improve the ability to discriminate the LNM status in EGC.

In recent days, CNN research on various malignancies is in its early stages, to reduce the stress of medical work and improve the utilization of medical resources through artificial intelligence technology (36–38). Several intelligent systems based on CT images or pathological images have been tested in GC employing deep learning network technology. With a sensitivity of near 100% and an average specificity of 80.6%, Song et al. developed a deep learning model to improve diagnostic accuracy and consistency of whole slide images of GC by automatic analysis (39). Two deep learning predictive models based on radiomics from two multicenter studies showed a good predictive value for LNM in GC with median AUCs of 0.876 (95% CI: 0.856–0.893) and 0.797 (95% CI: 0.771–0.823) in the external validation cohorts, respectively (23, 40). Due to the aforementioned research mainly focusing on advanced GC, it is difficult to apply these predictive models to EGC. In this study, we developed a model based on radiomics and DTL features to discriminate the LNM status in EGC with AUCs of 0.673 (95% CI: 0.580–0.766) and 0.581 (95% CI: 0.415–0.746) in the internal and external validation cohorts, respectively. The ability of the model was relatively lower than that of the aforementioned two types of research, while clinical features combined with this model showed significantly good performance to distinguish the LNM status with AUCs of 0.901 (95% CI: 0.847–0.956) and 0.915 (95% CI: 0.850–0.981) in the internal and external validation cohorts, respectively, which may be used to guide therapy options for individuals with EGC. For the unsatisfactory performance of radiomics and DTL features, there are the following reasons: first, the tumor size of EGC was relatively smaller than that of advanced GC, which limited the utilization of high-dimensional quantitative data of CT images; second, only the maximal ROI slice of the tumor was selected for DTL network analysis, and adding up-and-down- slices may improve the predictive performance. In addition, we found that the AUC value of random forest and XGBoost in various models was highest in the training cohort; however, the results of the internal validation and external validation cohorts were both insufficient. We speculated that the model was over-classified in the training cohort and represented too many branches, resulting in overfitting of the model.

In this study, the parameters of several deep learning networks were trained by maximal rectangular slice ROIs of EGC, including Resnet152, Resnet101, Resnet50, Resnet34, Resnet18, Wide_resnet101_2, Wide_resnet50_2, and Inception v3. Previous studies selected different deep learning networks used to build models with satisfactory performance, such as VGG-19, DenseNet-201, Resnet18, and Resnet50, so it is necessary to find a suitable pre-trained deep learning network for our research (17, 21, 23, 40). Resnet incorporates residual learning to prevent gradient dispersion and accuracy reduction in deep networks, resulting in increased network efficiency, accuracy, and execution speed (21). For example, Resnet152 is a 152-layer convolutional neural network, including convolutional layers and fully connected layers. The Inception module is distinguished by the fact that convolution cores of varying sizes are convolved on the same feature map, adding parallel pooling, and the results are aggregated as input for the next layer, which allows for the acquisition of a greater abundance of different size features (41). In all models we constructed, the pre-trained Resnet152 network had the best performance in discriminating the LNM status in EGC with AUCs of 0.901 (95% CI: 0.847–0.956) and 0.915 (95% CI: 0.850–0.981) in the internal and external validation cohorts, respectively. In addition, the pre-trained Inception v3 network also showed good ability with AUCs of 0.897 (95% CI: 0.844–0.950) and 0.900 (95% CI: 0.825–0.976) in two validation cohorts. However, the pre-trained Resnet18 showed relatively lower performance with AUCs of 0.831 (95% CI: 0.761–0.901) and 0.862 (95% CI: 0.762–0.963) in the internal and external validation cohorts, respectively. Thus, it is important to find a suitable CNN to improve the ability to diagnose in cancer research.

Artificial intelligence (AI) mainly includes two primary branches of deep learning and machine learning. It is a branch of computer science dedicated to creating a machine that models human cognitive capabilities, including learning and problem-solving. Single-center observational research was carried out to assess the effectiveness of CAD in the diagnosis of EGC utilizing magnifying endoscopy with narrow-band imaging. CAD system diagnostic performance was equivalent to the majority of experienced endoscopists compared to 11 professional endoscopists (42). Although the unsatisfactory performance of radiomics and DTL features model in our research, AI still has the potential to be valuable tool in cancer screening, diagnosis, and treatment with the development of the algorithm and the updating of technology. In addition, larger prospective trials examining the use of AI throughout the gastric cancer diagnosis and therapy are required to accurately assess its effectiveness and utility in clinical practice.

There are some limitations to this retrospective study. First, when inclusion and exclusion criteria were strict, the sample bias would have an impact on model training. Because of low CT imaging quality, 327 patients were excluded from this study. Second, the radiomics features were only extracted from CT images of the portal phase, and other phases of CT images may provide more important features. Third, larger prospective trials are necessary for evaluating the ability of the predictive model in clinical practice. Finally, two-dimensional segmentation may not be representative of the complete tumor, and some characteristics may be influenced by two-dimensional versus three-dimensional segmentation. However, in our DTL analysis, we only employed two-dimensional features from the maximal ROI slice of the tumor, instead of 3D features.

## Conclusion

We first integrated multi-model data based on clinical variables combining radiomics features with DTL features with a good predictive ability for discriminating the LNM status in EGC, which could provide favorable information for choosing individualized therapy options.

## Data availability statement

The original contributions presented in the study are included in the article/Supplementary material, further inquiries can be directed to the corresponding authors.

## Ethics statement

The studies involving human participants were reviewed and approved by the Ethics Committee of the First Affiliated Hospital of Nanchang University. Written informed consent for participation was not required for this study in accordance with the national legislation and the institutional requirements.

## Author contributions

QZ and ZF conceived the project and wrote the manuscript. YZ and FZ drew the ROI of CT images. XS, AW, and LL participated in data analysis. YC and YT participated in the discussion and language editing. JX and ZL reviewed the manuscript. HL provided an external validation cohort. All authors read and approved the final manuscript.

## Footnotes

1
https://www.python.org/

2
https://www.r-project.org/

## Supplementary material

Supplementary Datasheet 1 Original clinical features.

Supplementary Datasheet 2 Original radiomics features.

Supplementary Figure 1 The Area under the curve (AUC) of various groups of feature fusion in the training and external validation cohorts. **(A)** DTL features (Resnet152); **(B)** clinical features; **(C)** radiomics features; **(D)** DTL features (Resnet152) + clinical features; **(E)** clinical + radiomics features; **(F)** DTL features (Resnet152) + radiomics features. DTL, deep transfer learning.

Supplementary Figure 2 Performance of different machine learning classifications based on radiomics + DTL (Resnet152) + clinical features in the training and external validation cohorts. **(A)** Support vector machine (SVM); **(B)** K-Nearest Neighbor (KNN); **(C)** random decision forests; and **(D)** XGBoost.

Supplementary Table 1 Intraclass correlation coefficients (ICC) analysis.

Supplementary Table 2 Various models selected features.

Supplementary Table 3 Various models’ features dimension reduction.

Supplementary Table 4 The *P*-value results of the Delong test between different models.

Supplementary Table 5 The different performance of various classification models and the performance of various classifier in different deep learning models.

Supplementary Table 6 Original deep transfer learning features.

## Abbreviations

LNM: lymph node metastasis
EGC: early gastric cancer
DTL: deep transfer learning features
LASSO: least absolute shrinkage and selection operator
SVM: support vector machine
KNN: K-nearest neighbor
RF: random decision forest
CT: computed tomography
CNNs: convolutional neural networks
ESD: endoscopic submucosal dissection
ROI: region of interest
ICC: intra-/inter-class correlation coefficient
3D: three-dimensional.
