# Supplementary figures and images for: Development and validation of a predictive model combining clinical, radiomics, and deep transfer learning features for lymph node metastasis in early gastric cancer
Source: Front Med (Lausanne). 2022 Oct 3;9:986437. doi: 10.3389/fmed.2022.986437 (PMC9573999; doi:10.3389/fmed.2022.986437)

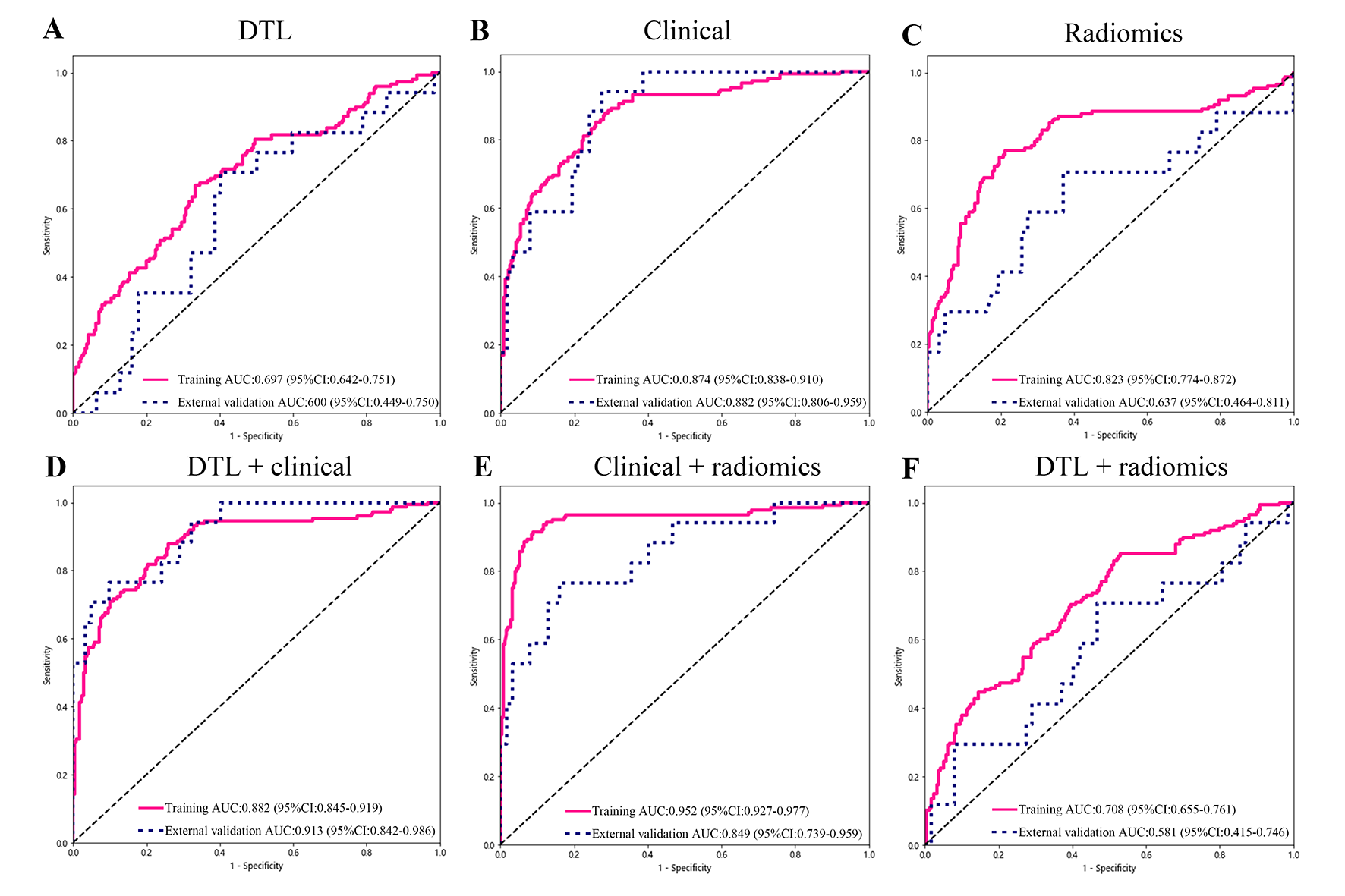

Supplement: Supplementary Figure 1 — The Area under the curve (AUC) of various groups of feature fusion in the training and external validation cohorts. (A) DTL features (Resnet152); (B) clinical features; (C) radiomics features; (D) DTL features (Resnet152) + clinical features; (E) clinical + radiomics features; (F) DTL features (Resnet152) + radiomics features. DTL, deep transfer learning. [file Image_1.TIF]

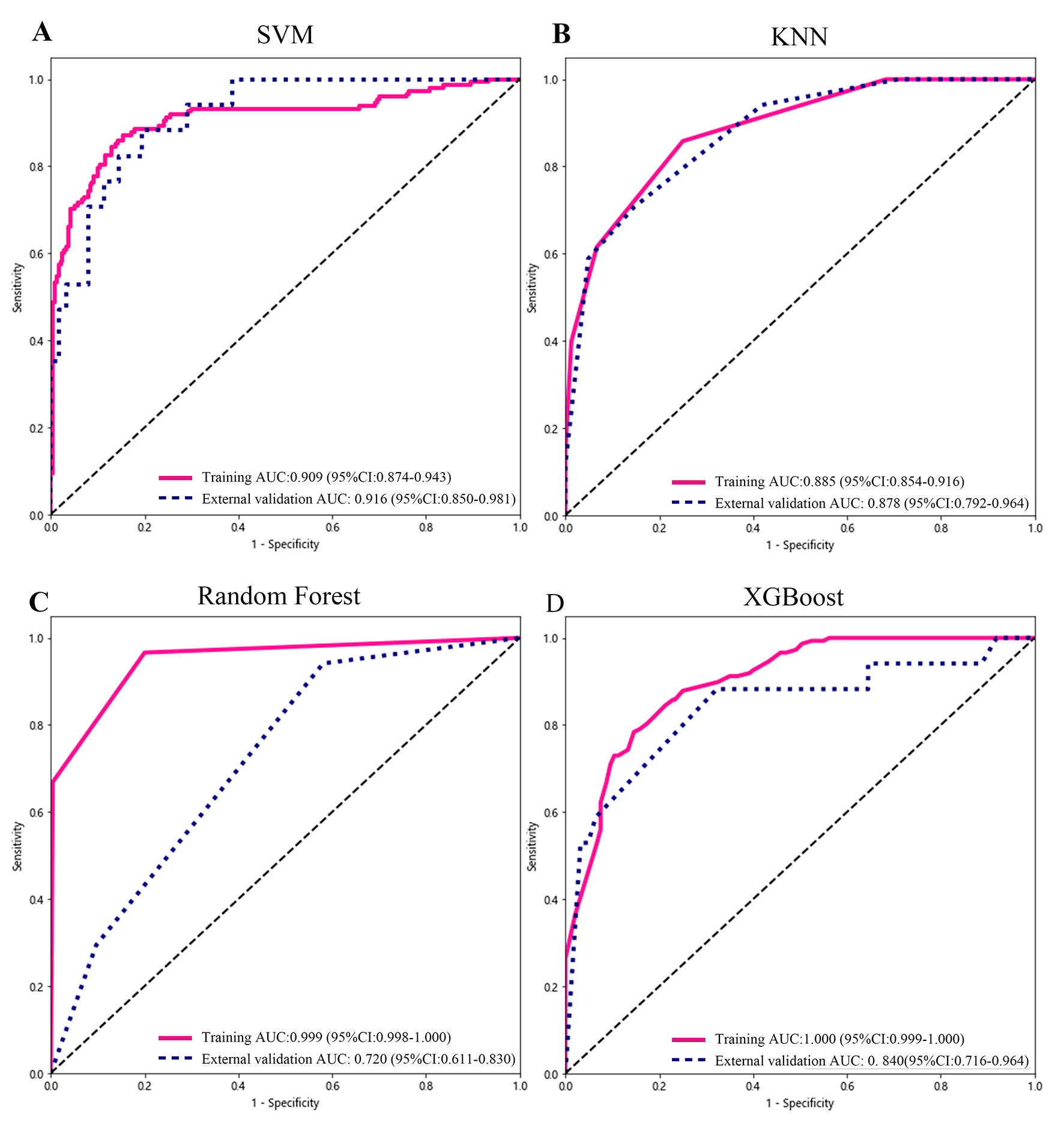

Supplement: Supplementary Figure 2 — Performance of different machine learning classifications based on radiomics + DTL (Resnet152) + clinical features in the training and external validation cohorts. (A) Support vector machine (SVM); (B) K-Nearest Neighbor (KNN); (C) random decision forests; and (D) XGBoost. [file Image_2.TIF]
