# Supplementary material for: Development and validation of a predictive model combining clinical, radiomics, and deep transfer learning features for lymph node metastasis in early gastric cancer
Source: Front Med (Lausanne). 2022 Oct 3;9:986437. doi: 10.3389/fmed.2022.986437 (PMC9573999; doi:10.3389/fmed.2022.986437)
Supplement: Supplementary Table 3 — Various models’ features dimension reduction. [file Table_3.DOCX]

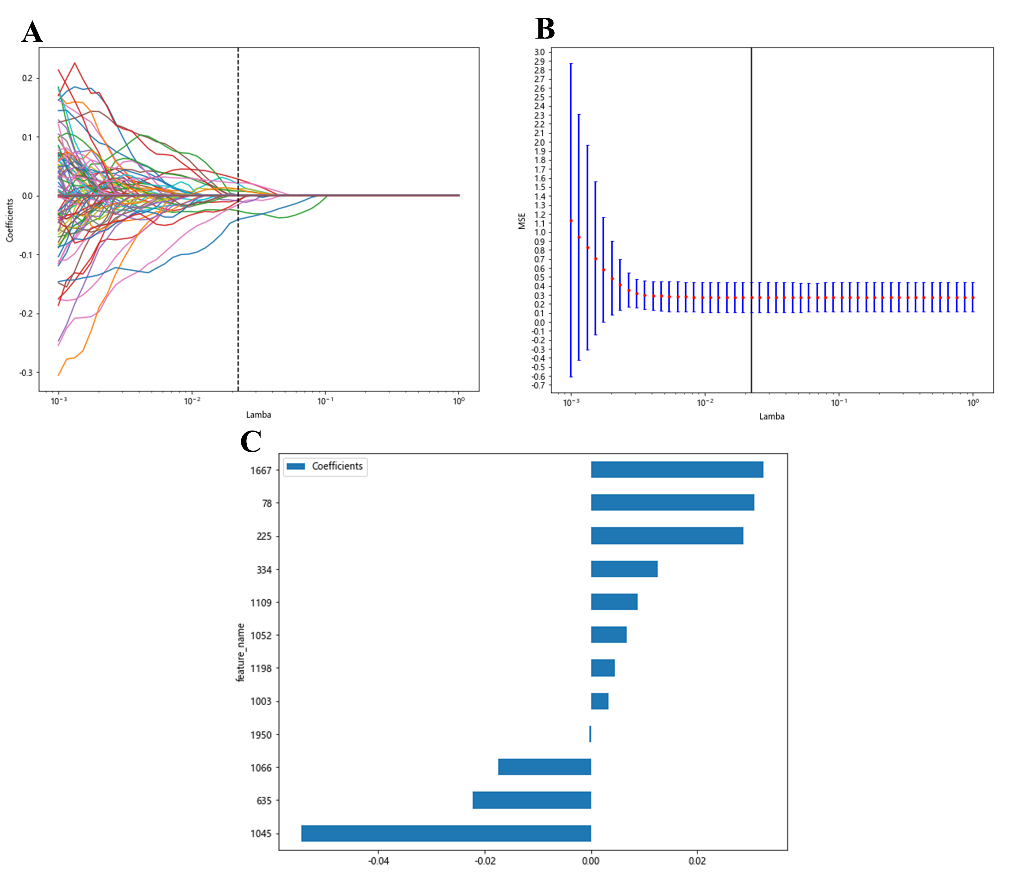
**Supplement material**


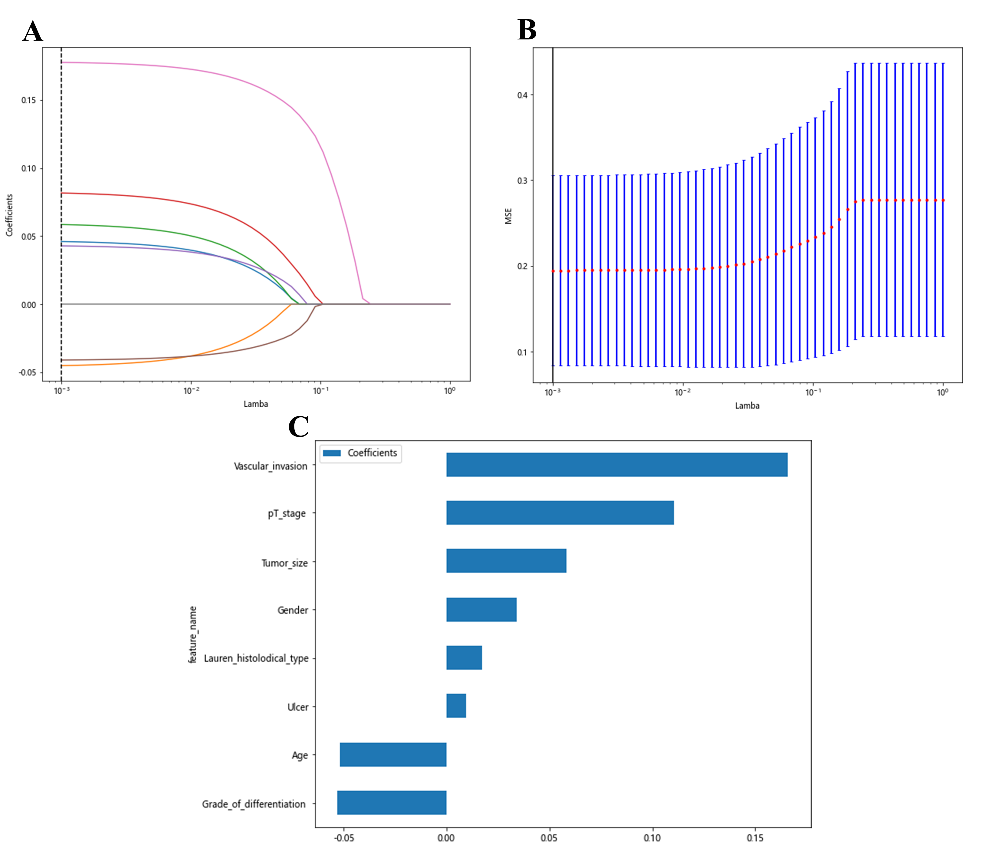
**Figure S1.** Deep transfer learning (DTL, Resnet152) network features dimension reduction.

**Figure S2.** Clinical features dimension reduction.


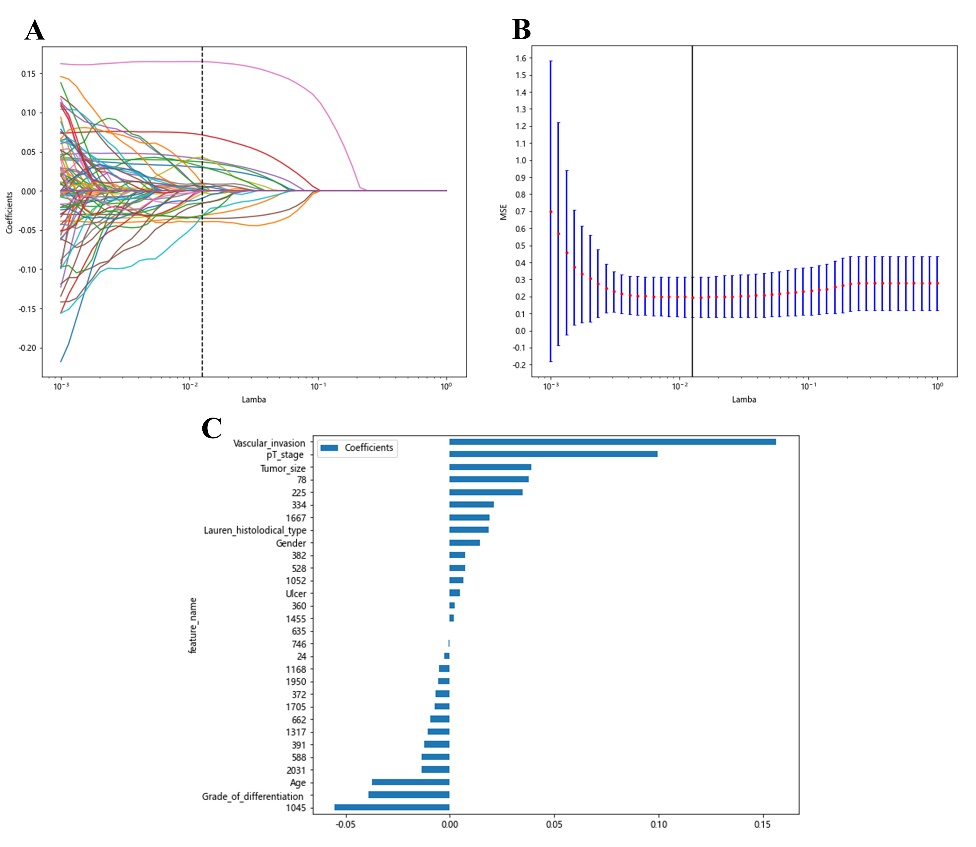

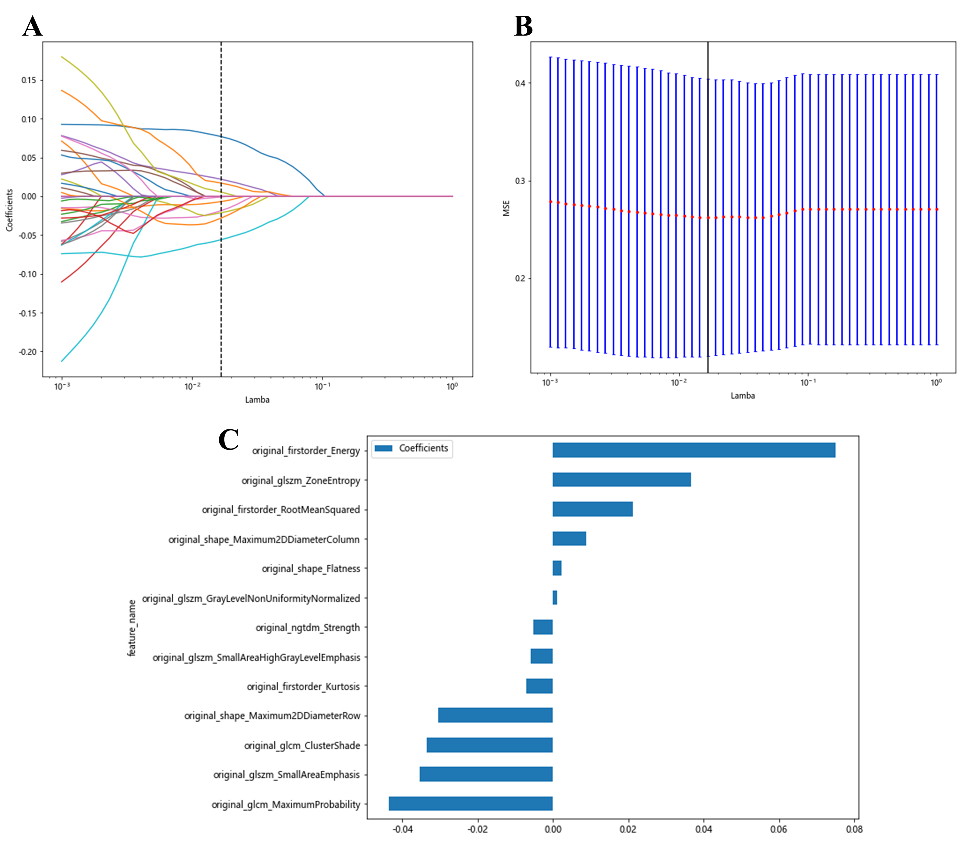
**Figure S3.** Radiomics features dimension reduction.

**Figure S4.** DTL + clinical features dimension reduction.


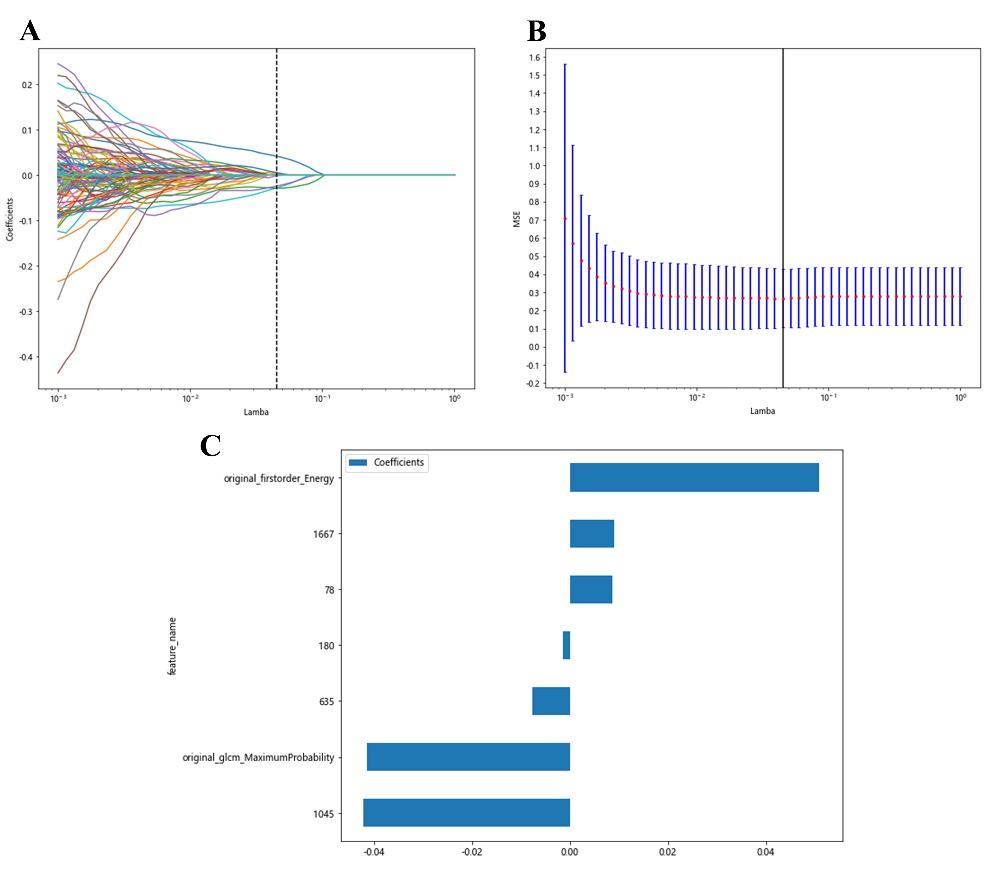

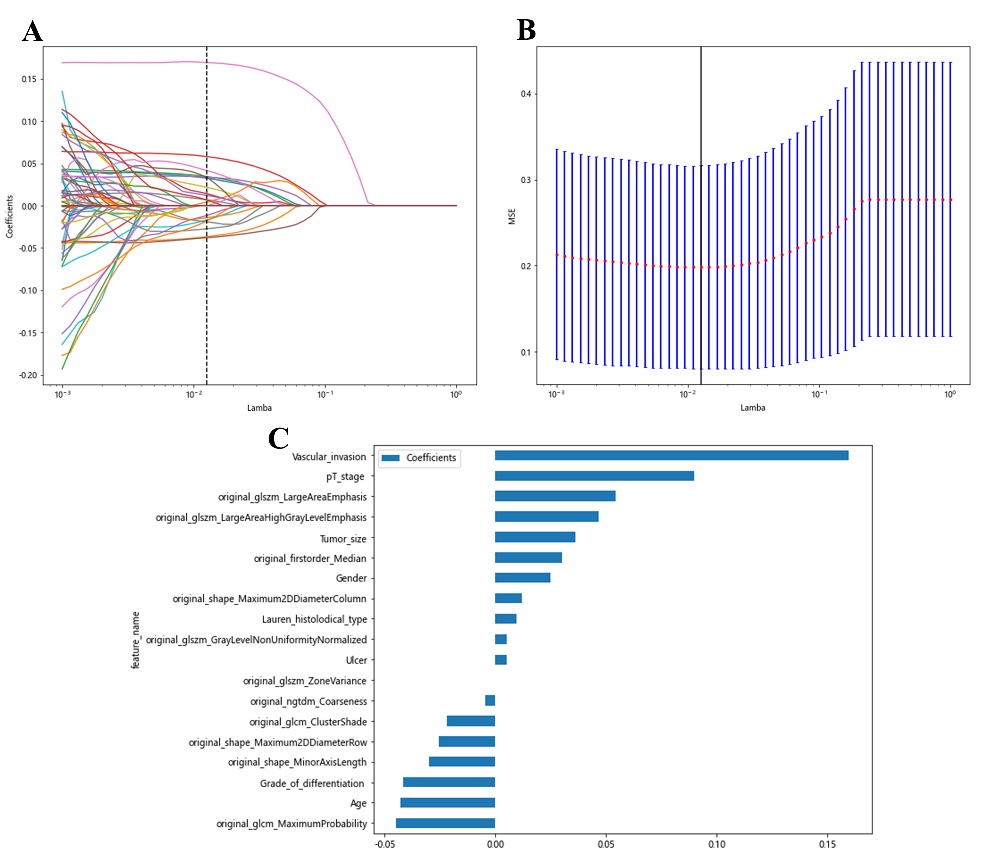
**Figure S5.** Radiomics + clinical features dimension reduction.

**Figure S6.** Radiomics + DTL features dimension reduction.


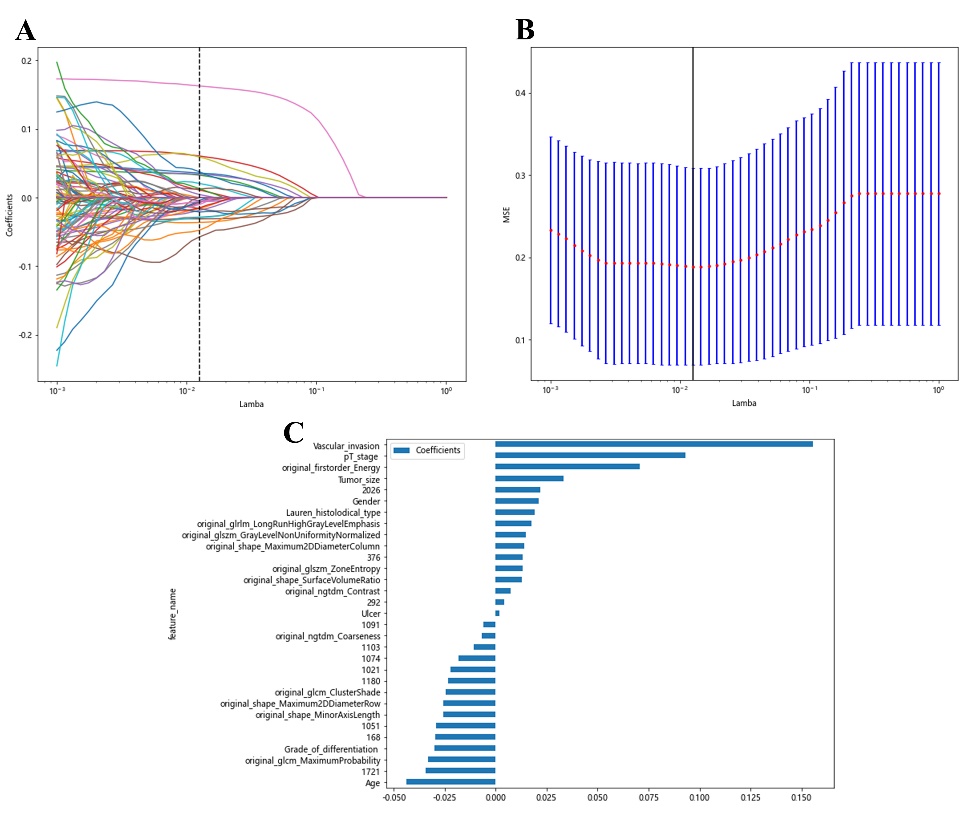

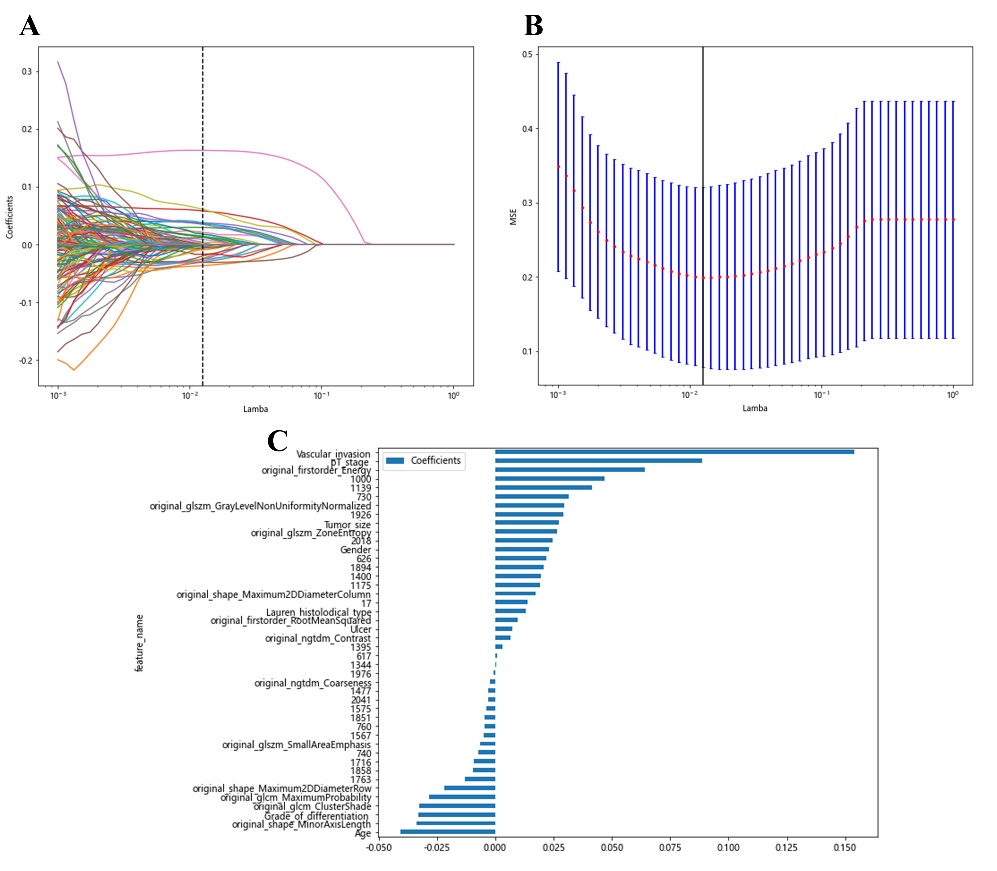
**Figure S7.** Radiomics + DTL(Resnet101) +clinical features dimension reduction.

**Figure S8.** Radiomics + DTL(Resnet50) +clinical features dimension reduction.


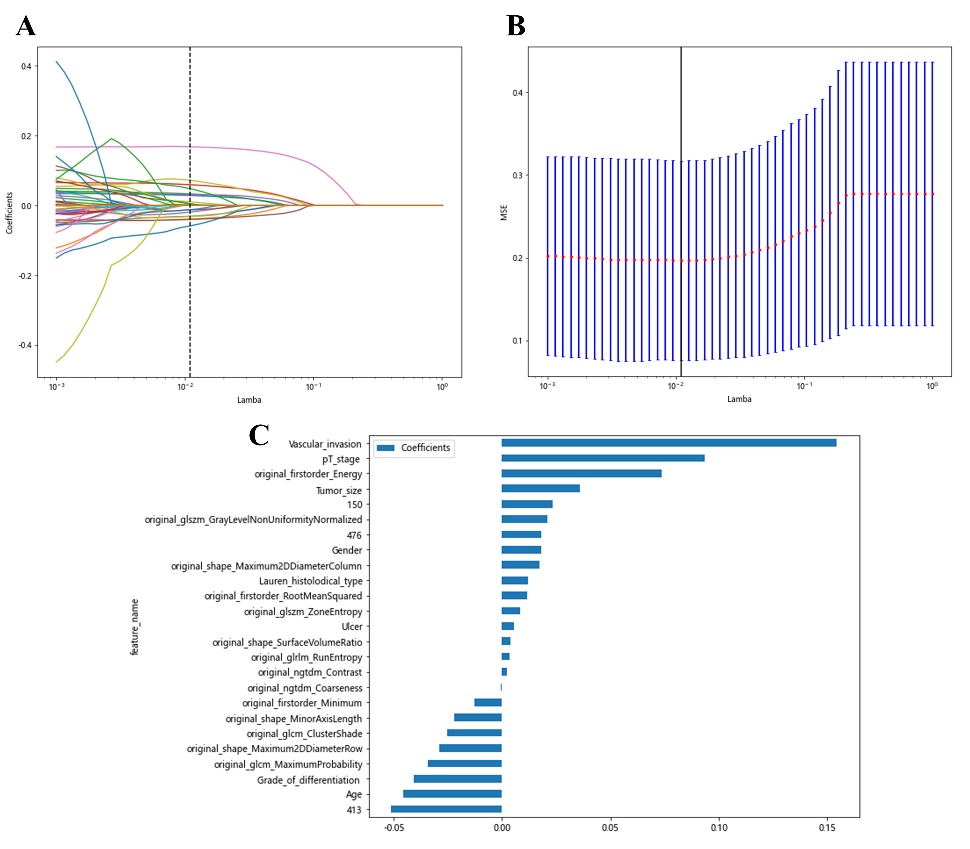

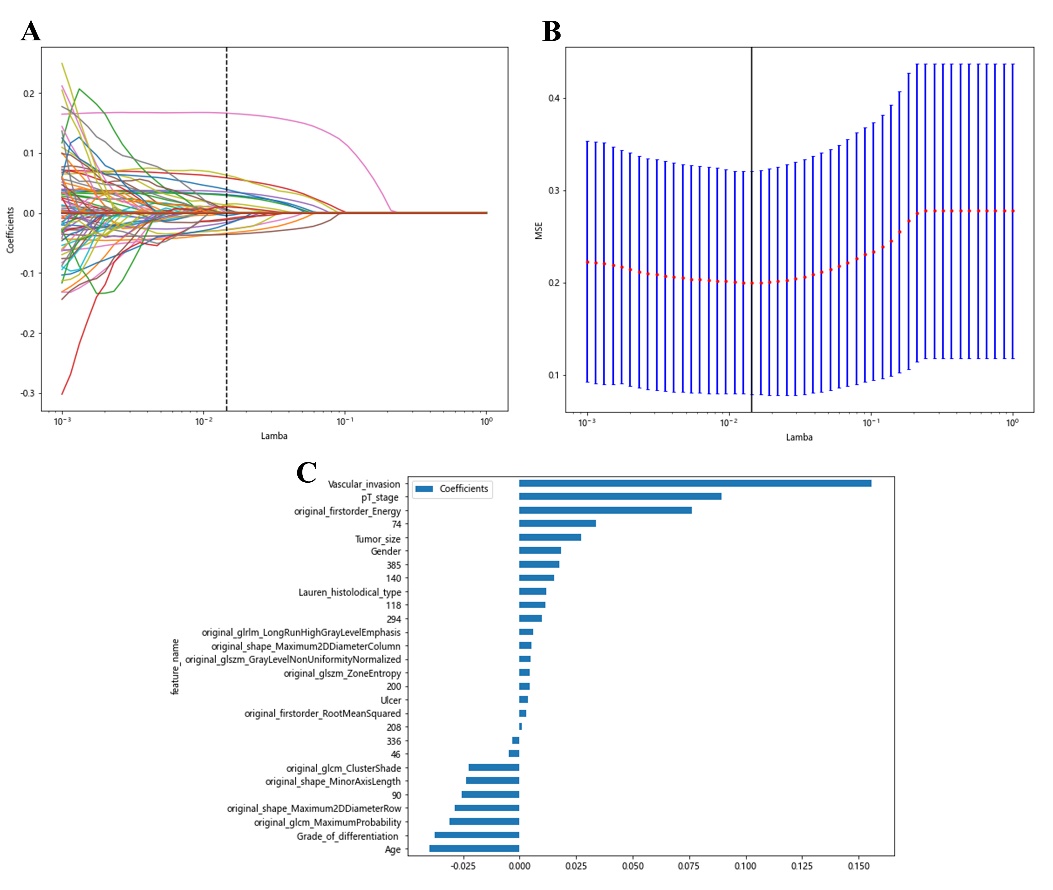
**Figure S9.** Radiomics + DTL(Resnet34) +clinical features dimension reduction.

**Figure S10.** Radiomics + DTL(Resnet18) +clinical features dimension reduction.

**
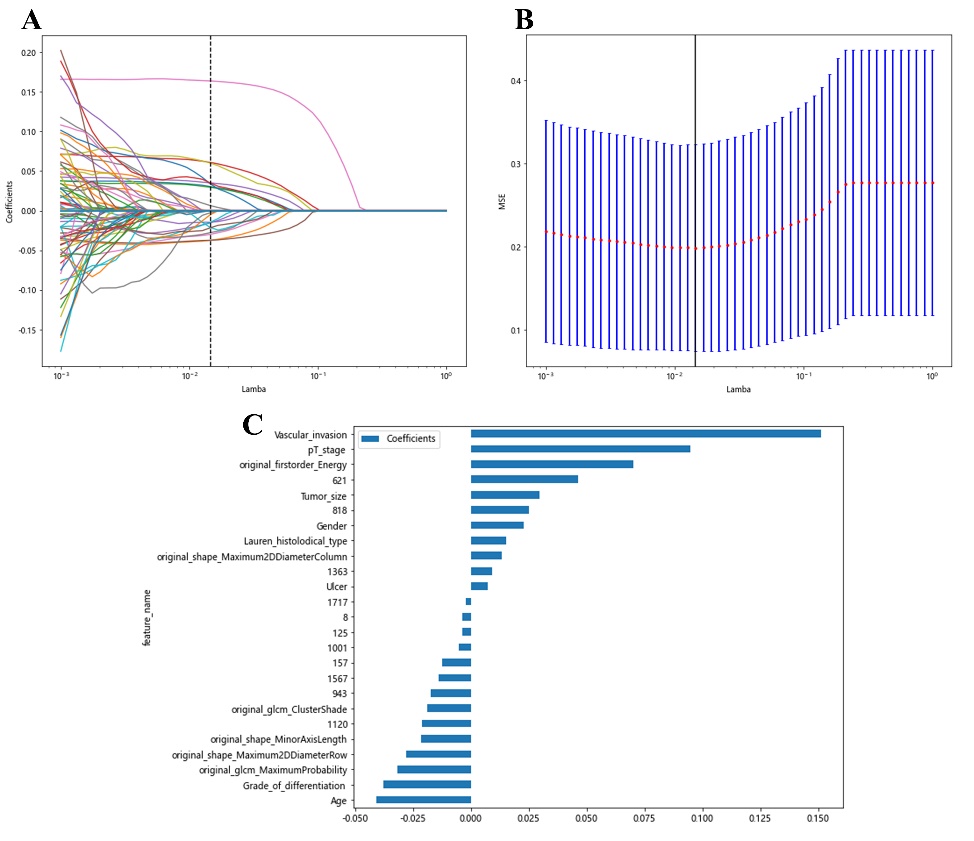
**
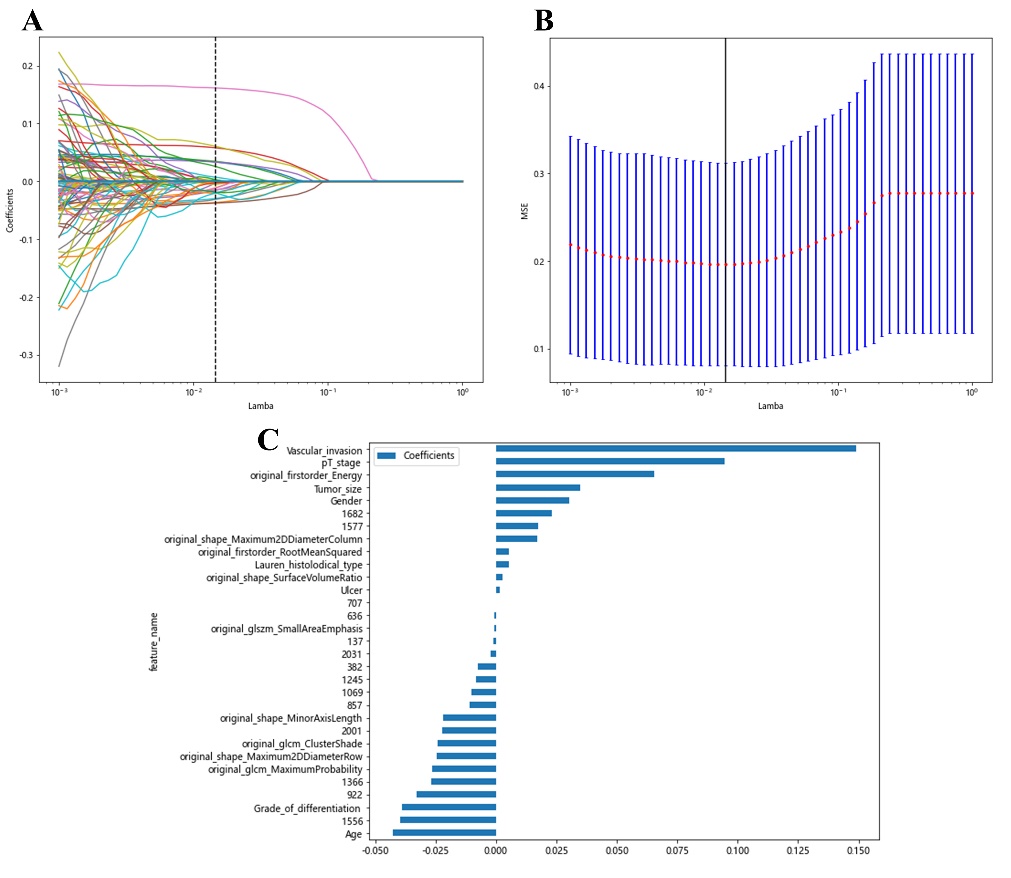
**Figure S11.** Radiomics + DTL(wide_resnet101_2) +clinical features dimension reduction.

**Figure S12.** Radiomics + DTL(wide_resnet50_2) +clinical features dimension reduction

**
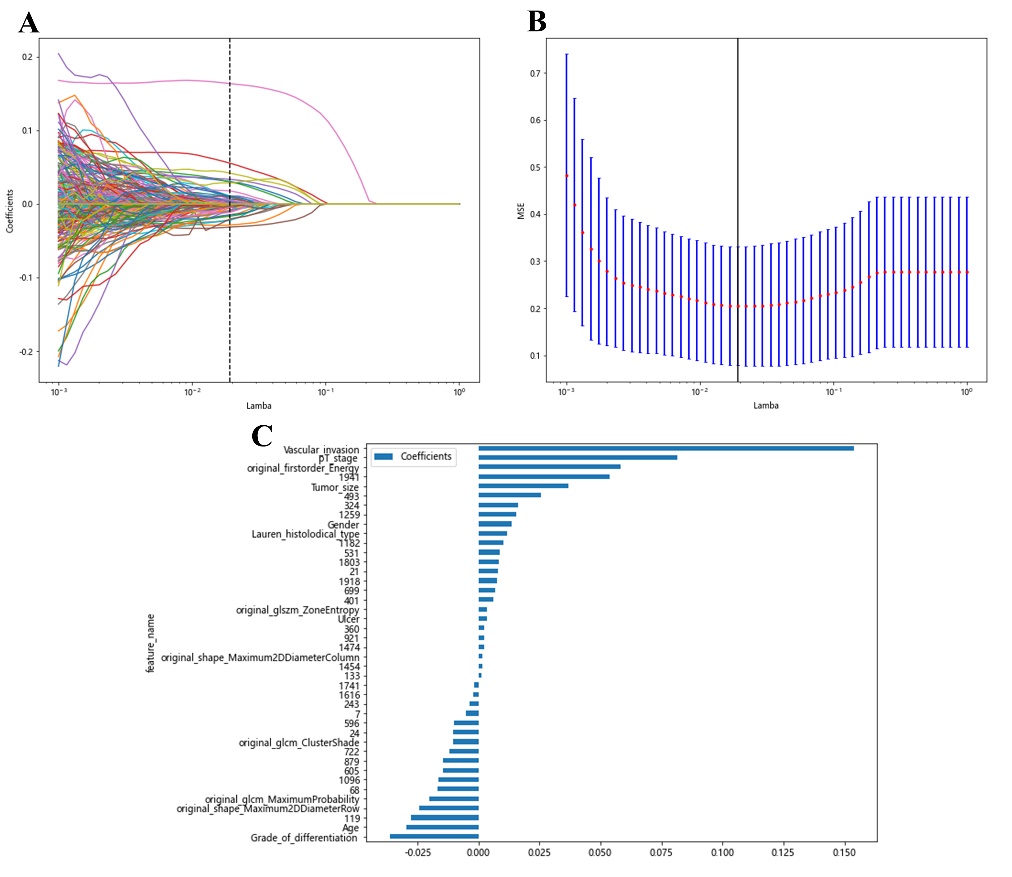
Figure S13.** Radiomics + DTL (Inception V3) +clinical features dimension reduction
