# Supplementary material for: Development and validation of a predictive model combining clinical, radiomics, and deep transfer learning features for lymph node metastasis in early gastric cancer
Source: Front Med (Lausanne). 2022 Oct 3;9:986437. doi: 10.3389/fmed.2022.986437 (PMC9573999; doi:10.3389/fmed.2022.986437)
Supplement: Supplementary Table 5 — The different performance of various classification models and the performance of various classifier in different deep learning models. [file Table_5.DOCX]

**Supplement material**

Table S2 The different performance of various classification models

| **Group** | **Classifier** | **Cohorts** | **AUC (95%CI)** | **Accuracy** | **Sensitivity** | **Specificity** |
| --- | --- | --- | --- | --- | --- | --- |
| **DTL features** | SVM | Training | 0.697(0.642-0.751) | 0.660 | 0.669 | 0.667 |
|  |  | Internal validation | 0.687(0.600-0.773) | 0.725 | 0.857 | 0.476 |
|  |  | External validation | 0.600(0.450-0.750) | 0.785 | 0.706 | 0.607 |
|  | KNN | Training | 0.837(0.800-0.875) | 0.760 | 0.851 | 0.667 |
|  |  | Internal validation | 0.589(0.497-0.681) | 0.640 | 0.693 | 0.517 |
|  |  | External validation | 0.675(0.546-0.805) | 0.696 | 0.764 | 0.508 |
|  | RF | Training | 0.996(0.994-0.999) | 0.961 | 0.984 | 0.975 |
|  |  | Internal validation | 0.608(0.515-0.702) | 0.677 | 0.428 | 0.786 |
|  |  | External validation | 0.668(0.523-0.813) | 0.696 | 0.647 | 1.000 |
|  | XGBoost | Training | 0.962(0.945-0.979) | 0.899 | 0.905 | 0.917 |
|  |  | Internal validation | 0524(0.431-0.616) | 0.568 | 0.571 | 0.556 |
|  |  | External validation | 0.804(0.713-0.895) | 0.785 | 0.882 | 0.705 |
| **Clinical variables** | SVM | Training | 0.874(0.838-0.910) | 0.794 | 0.878 | 0.717 |
|  |  | Internal validation | 0.807(0.731-0.884) | 0.796 | 0.735 | 0.805 |
|  |  | External validation | 0.882(0.806-0.960) | 0.785 | 0.941 | 0.726 |
|  | KNN | Training | 0.865(0.830-0.899) | 0.809 | 0.648 | 0.908 |
|  |  | Internal validation | 0.746(0.666-0.826) | 0.731 | 0.449 | 0.956 |
|  |  | External validation | 0.837(0.748-0.925) | 0.709 | 0.882 | 0.695 |
|  | RF | Training | 0.998(0.996-1.000) | 0.979 | 1.000 | 0.967 |
|  |  | Internal validation | 0.788(0.709-0.867) | 0.784 | 0.714 | 0.828 |
|  |  | External validation | 0.670(0.534-0.805) | 0.709 | 0.765 | 0.694 |
|  | XGBoost | Training | 0.917(0.890-0.944) | 0.835 | 0.858 | 0.825 |
|  |  | Internal validation | 0.782(0.695-0.869) | 0.772 | 0.633 | 0.907 |
|  |  | External validation | 0.796(0.655-0.938) | 0.785 | 0.588 | 0.952 |
| **Radiomics features** | SVM | Training | 0.809(0.761-0.858) | 0.724 | 0.770 | 0.788 |
|  |  | Internal validation | 0.631(0.540-0.724) | 0.689 | 0.776 | 0.530 |
|  |  | External validation | 0.637(0.464-0.811) | 0.747 | 0.706 | 0.629 |
|  | KNN | Training | 0.788(0.746-0.830) | 0.739 | 0.858 | 0.554 |
|  |  | Internal validation | 0.587(0.495-0.679) | 0.658 | 0.653 | 0.482 |
|  |  | External validation | 0.595(0.438-0.751) | 0.671 | 0.471 | 0.763 |
|  | RF | Training | 0.998(0.997-1.000) | 0.987 | 0.986 | 0.987 |
|  |  | Internal validation | 0.530(0.433-0.627) | 0.592 | 0.632 | 0.444 |
|  |  | External validation | 0.499(0.364-0.634) | 0.709 | 0.647 | 0.436 |
|  | XGBoost | Training | 0.996(0.994-0.999) | 0.963 | 0.972 | 0.970 |
|  |  | Internal validation | 0.560(0.464-0.655) | 0.622 | 0.816 | 0.316 |
|  |  | External validation | 0.537(0.358-0.717) | 0.785 | 0.353 | 0.919 |
| **DTL features + Clinical variables** | SVM | Training | 0.882(0.845-0.919) | 0.789 | 0.878 | 0.742 |
|  |  | Internal validation | 0.878(0.819-0.937) | 0.814 | 0.857 | 0.797 |
|  |  | External validation | 0.914(0.842-0.986) | 0.886 | 0.765 | 0.903 |
|  | KNN | Training | 0.887(0.856-0.918) | 0.822 | 0.668 | 0.917 |
|  |  | Internal validation | 0.764(0.682-0.846) | 0.772 | 0.633 | 0.852 |
|  |  | External validation | 0.846(0.746-0.946) | 0.772 | 0.529 | 1.000 |
|  | RF | Training | 0.999(0.999-1.000) | 0.992 | 0.979 | 1.000 |
|  |  | Internal validation | 0.801(0.728-0.874) | 0.706 | 0.857 | 0.650 |
|  |  | External validation | 0.697(0.565-0.829) | 0.797 | 0.824 | 0.500 |
|  | XGBoost | Training | 0.998(0.995-1.000) | 0.982 | 0.979 | 0.995 |
|  |  | Internal validation | 0.810(0.740-0.881) | 0.748 | 0.897 | 0.627 |
|  |  | External validation | 0.774(0.632-0.915) | 0.873 | 0.588 | 0.983 |
| **Clinical variables + Radiomics features** | SVM | Training | 0.952(0.927-0.977) | 0.822 | 0.900 | 0.915 |
|  |  | Internal validation | 0.844(0.780-0.910) | 0.808 | 0.673 | 0.873 |
|  |  | External validation | 0.849(0.739-0.959) | 0.835 | 0.765 | 0.839 |
|  | KNN | Training | 0.876(0.843-0.908) | 0.799 | 0.842 | 0.754 |
|  |  | Internal validation | 0.731(0.640-0.822) | 0.754 | 0.510 | 0.856 |
|  |  | External validation | 0.692(0.556-0.828) | 0.709 | 0.765 | 0.567 |
|  | RF | Training | 0.998(0.997-1.000) | 0.992 | 0.986 | 0.995 |
|  |  | Internal validation | 0.749(0.668-0.829) | 0.742 | 0.857 | 0.568 |
|  |  | External validation | 0.752(0.624-0.881) | 0.835 | 0.824 | 0.567 |
|  | XGBoost | Training | 0.999(0.999-1.000) | 0.992 | 0.992 | 1.000 |
|  |  | Internal validation | 0.763(0.678-0.848) | 0.766 | 0.653 | 0.813 |
|  |  | External validation | 0.803(0.681-0.926) | 0.785 | 0.706 | 0.867 |
| **DTL features + Radiomics features** | SVM | Training | 0.707(0.655-0.761) | 0.660 | 0.851 | 0.471 |
|  |  | Internal validation | 0.673(0.580-0.766) | 0.719 | 0.694 | 0.619 |
|  |  | External validation | 0.581(0.415-0.746) | 0.760 | 0.706 | 0.541 |
|  | KNN | Training | 0.810(0.769-0.852) | 0.757 | 0.851 | 0.608 |
|  |  | Internal validation | 0.633(0.546-0.720) | 0.652 | 0.734 | 0.491 |
|  |  | External validation | 0.630(0.498-0.763) | 0.658 | 0.706 | 0.517 |
|  | RF | Training | 0.998(0.996-1.000) | 0.974 | 0.979 | 0.983 |
|  |  | Internal validation | 0.593(0.501-0.685) | 0.658 | 0.734 | 0.452 |
|  |  | External validation | 0.692(0.586-0.797) | 0.734 | 0.941 | 0.509 |
|  | XGBoost | Training | 0.960(0.941-0.979) | 0.884 | 0.905 | 0.925 |
|  |  | Internal validation | 0.658(0.568-0.748) | 0.670 | 0.775 | 0.508 |
|  |  | External validation | 0.633(0.515-0.752) | 0.785 | 0.824 | 0.541 |
| **DTL features + Clinical variables + Radiomics features** | SVM | Training | 0.909(0.874-0.943) | 0.830 | 0.872 | 0.846 |
|  |  | Internal validation | 0.901(0.847-0.956) | 0.962 | 0.800 | 0.881 |
|  |  | External validation | 0.916(0.850-0.981) | 0.861 | 0.882 | 0.806 |
|  | KNN | Training | 0.885(0.854-0.916) | 0.812 | 0.858 | 0.750 |
|  |  | Internal validation | 0.793(0.712-0.874) | 0.790 | 0.857 | 0.602 |
|  |  | External validation | 0.878(0.792-0.964) | 0.823 | 0.706 | 0.855 |
|  | RF | Training | 0.999(0.998-1.000) | 0.989 | 0.993 | 0.983 |
|  |  | Internal validation | 0.810(0.741-0.879) | 0.748 | 0.918 | 0.635 |
|  |  | External validation | 0.720(0.611-0.830) | 0.772 | 0.941 | 0.464 |
|  | XGBoost | Training | 0.999(0.999-1.000) | 0.987 | 1.000 | 0.991 |
|  |  | Internal validation | 0.820(0.741-0.900) | 0.808 | 0.755 | 0.839 |
|  |  | External validation | 0.840(0.716-0.964) | 0.873 | 0.882 | 0.700 |

Table S3 The performance of various classifier in different deep learning models

| **Group** | **Classifier** | **Cohorts** | **AUC (95%CI)** | **Accuracy** | **Sensitivity** | **Specificity** |
| --- | --- | --- | --- | --- | --- | --- |
| **Resnet152** | SVM | Training | 0.909(0.874-0.943) | 0.830 | 0.872 | 0.846 |
|  |  | Internal validation | 0.901(0.847-0.956) | 0.962 | 0.800 | 0.881 |
|  |  | External validation | 0.916(0.850-0.981) | 0.861 | 0.882 | 0.806 |
|  | KNN | Training | 0.885(0.854-0.916) | 0.812 | 0.858 | 0.750 |
|  |  | Internal validation | 0.793(0.712-0.874) | 0.790 | 0.857 | 0.602 |
|  |  | External validation | 0.878(0.792-0.964) | 0.823 | 0.706 | 0.855 |
|  | RF | Training | 0.999(0.998-1.000) | 0.989 | 0.993 | 0.983 |
|  |  | Internal validation | 0.810(0.741-0.879) | 0.748 | 0.918 | 0.635 |
|  |  | External validation | 0.720(0.611-0.830) | 0.772 | 0.941 | 0.464 |
|  | XGBoost | Training | 0.999(0.999-1.000) | 0.987 | 1.000 | 0.991 |
|  |  | Internal validation | 0.820(0.741-0.900) | 0.808 | 0.755 | 0.839 |
|  |  | External validation | 0.840(0.716-0.964) | 0.873 | 0.882 | 0.700 |
| **Resnet101** | SVM | Training | 0.923(0.889-0.958) | 0.835 | 0.885 | 0.904 |
|  |  | Internal validation | 0.887(0.829-0.945) | 0.826 | 0.735 | 0.898 |
|  |  | External validation | 0.899(0.803-0.996) | 0.848 | 0.882 | 0.839 |
|  | KNN | Training | 0.855(0.819-0.891) | 0.804 | 0.587 | 0.937 |
|  |  | Internal validation | 0.739(0.651-0.827) | 0.760 | 0.551 | 0.847 |
|  |  | External validation | 0.869(0.783-0.954) | 0.824 | 0.806 | 0.600 |
|  | RF | Training | 0.999(0.998-1.000) | 0.984 | 0.986 | 0.987 |
|  |  | Internal validation | 0.760(0.679-0.841) | 0.772 | 0.571 | 0.820 |
|  |  | External validation | 0.705(0.590-0.820) | 0.671 | 0.941 | 0.636 |
|  | XGBoost | Training | 1.000 | 1.000 | 1.000 | 1.000 |
|  |  | Internal validation | 0.823(0.748-0.898) | 0.772 | 0.775 | 0.788 |
|  |  | External validation | 0.869(0.776-0.963) | 0.861 | 0.941 | 0.629 |
| **Resnet50** | SVM | Training | 0.937(0.909-0.966) | 0.869 | 0.892 | 0.892 |
|  |  | Internal validation | 0.882(0.825-0.940) | 0.850 | 0.735 | 0.898 |
|  |  | External validation | 0.900(0.821-0.980) | 0.823 | 0.941 | 0.790 |
|  | KNN | Training | 0.884(0.853-0.915) | 0.791 | 0.885 | 0.754 |
|  |  | Internal validation | 0.771(0.681-0.860) | 0.802 | 0.612 | 0.881 |
|  |  | External validation | 0.761(0.615-0.907) | 0747 | 0.471 | 0.968 |
|  | RF | Training | 0.999(0.998-1.000) | 0.984 | 0.979 | 0.995 |
|  |  | Internal validation | 0.820(0.741-0.898) | 0.808 | 0.632 | 0.881 |
|  |  | External validation | 0.629(0.462-0.795) | 0.797 | 0.471 | 1.000 |
|  | XGBoost | Training | 0.999(0.999-1.000) | 0.992 | 1.000 | 0.996 |
|  |  | Internal validation | 0.834(0.758-0.909) | 0.802 | 0.734 | 0.831 |
|  |  | External validation | 0.849(0.731-0.967) | 0.785 | 0.765 | 0.855 |
| **Resnet34** | SVM | Training | 0.916(0.883-0.949) | 0.832 | 0.872 | 0.858 |
|  |  | Internal validation | 0.877(0.817-0.937) | 0.832 | 0.755 | 0.864 |
|  |  | External validation | 0.884(0.805-0.963) | 0.823 | 0.941 | 0.694 |
|  | KNN | Training | 0.887(0.845-0.910) | 0.807 | 0.858 | 0.738 |
|  |  | Internal validation | 0.775(0.692-0.859) | 0.766 | 0.449 | 0.976 |
|  |  | External validation | 0.883(0.798-0.967) | 0.835 | 0.765 | 0.855 |
|  | RF | Training | 0.999(0.998-1.000) | 0.985 | 0.986 | 0.992 |
|  |  | Internal validation | 0.820(0.747-0.879) | 0.796 | 0.755 | 0.777 |
|  |  | External validation | 0.904(0.832-0.975) | 0.911 | 0.765 | 1.000 |
|  | XGBoost | Training | 0.999(0.999-1.000) | 0.992 | 1.000 | 0.992 |
|  |  | Internal validation | 0.825(0.746-0.903) | 0.814 | 0.775 | 0.839 |
|  |  | External validation | 0.829(0.702-0.955) | 0.785 | 0.588 | 0.984 |
| **Resnet18** | SVM | Training | 0.939(0.911-0.967) | 0.838 | 0.919 | 0.879 |
|  |  | Internal validation | 0.831(0.761-0.901) | 0.820 | 0.592 | 0.932 |
|  |  | External validation | 0.862(0.762-0.963) | 0.810 | 0.824 | 0.806 |
|  | KNN | Training | 0.894(0.863-0.923) | 0.825 | 0.905 | 0.700 |
|  |  | Internal validation | 0.722(0.630-0.814) | 0.719 | 0.469 | 0.941 |
|  |  | External validation | 0.846(0.760-0.932) | 0.772 | 0.824 | 0.770 |
|  | RF | Training | 0.999(0.997-1.000) | 0.989 | 0.986 | 0.991 |
|  |  | Internal validation | 0.813(0.736-0.890) | 0.784 | 0.735 | 0.771 |
|  |  | External validation | 0.654(0.521-0.787) | 0.709 | 0.882 | 0.429 |
|  | XGBoost | Training | 0.998(0.997-1.000) | 0.976 | 1.000 | 0.966 |
|  |  | Internal validation | 0.826(0.745-0.908) | 0.808 | 0.795 | 0.822 |
|  |  | External validation | 0.771(0.606-0.938) | 0.785 | 0.588 | 0.952 |
| **Wide_resnet101_2** | SVM | Training | 0.921(0.889-0.954) | 0.835 | 0.865 | 0.892 |
|  |  | Internal validation | 0.859(0.795-0.922) | 0.826 | 0.755 | 0.881 |
|  |  | External validation | 0.846(0.742-0.951) | 0.747 | 0.882 | 0.661 |
|  | KNN | Training | 0.859(0.824-0.894) | 0.791 | 0.871 | 0.675 |
|  |  | Internal validation | 0.799(0.720-0.879) | 0.790 | 0.653 | 0.862 |
|  |  | External validation | 0.822(0.717-0.926) | 0.722 | 0.706 | 0.738 |
|  | RF | Training | 0.999(0.997-1.000) | 0.989 | 0.972 | 1.000 |
|  |  | Internal validation | 0.800(0.721-0.879) | 0.802 | 0.673 | 0.822 |
|  |  | External validation | 0.625(0.481-0.769) | 0.734 | 0.706 | 0.585 |
|  | XGBoost | Training | 0.999(0.999-1.000) | 0.984 | 0.993 | 0.996 |
|  |  | Internal validation | 0.834(0.763-0.905) | 0.778 | 0.673 | 0.873 |
|  |  | External validation | 0.866(0.762-0.971) | 0.785 | 0.882 | 0.754 |
| **Wide_resnet50_2** | SVM | Training | 0.937(0.909-0.964) | 0.840 | 0.851 | 0.896 |
|  |  | Internal validation | 0.868(0.806-0.929) | 0.844 | 0.714 | 0.907 |
|  |  | External validation | 0.888(0.800-0.976) | 0.861 | 0.706 | 0.903 |
|  | KNN | Training | 0.881(0.848-0.912) | 0.814 | 0.858 | 0.720 |
|  |  | Internal validation | 0.764(0.679-0.849) | 0.766 | 0.795 | 0.606 |
|  |  | External validation | 0.827(0.710-0.944) | 0.760 | 0.647 | 0.902 |
|  | RF | Training | 0.999(0.999-1.000) | 0.984 | 0.993 | 0.987 |
|  |  | Internal validation | 0.841(0.779-0.903) | 0.796 | 0.857 | 0.652 |
|  |  | External validation | 0.739(0.615-0.864) | 0.797 | 0.529 | 1.000 |
|  | XGBoost | Training | 0.999(0.998-1.000) | 0.971 | 0.986 | 0.996 |
|  |  | Internal validation | 0.846(0.777-0.914) | 0.790 | 0.755 | 0.805 |
|  |  | External validation | 0.819(0.689-0.950) | 0.861 | 0.706 | 0.885 |
| **Inception v3** | SVM | Training | 0.890(0.852-0.929) | 0.830 | 0.851 | 0.846 |
|  |  | Internal validation | 0.897(0.844-0.950) | 0.826 | 0.837 | 0.839 |
|  |  | External validation | 0.900(0.825-0.976) | 0.823 | 0.765 | 0.887 |
|  | KNN | Training | 0.858(0.823-0.892) | 0.771 | 0.871 | 0.683 |
|  |  | Internal validation | 0.846(0.776-0.915) | 0.826 | 0.694 | 0.889 |
|  |  | External validation | 0.872(0.771-0.974) | 0.835 | 0.824 | 0.852 |
|  | RF | Training | 0.999(0.998-1.000) | 0.989 | 0.993 | 0.995 |
|  |  | Internal validation | 0.818(0.748-0.887) | 0.784 | 0.612 | 0.855 |
|  |  | External validation | 0.689(0.554-0.825) | 0.734 | 0.529 | 1.000 |
|  | XGBoost | Training | 0.998(0.997-1.000) | 0.977 | 0.993 | 0.971 |
|  |  | Internal validation | 0.841(0.769-0.913) | 0.814 | 0.673 | 0.889 |
|  |  | External validation | 0.782(0.656-0.908) | 0.785 | 0.529 | 0.967 |

SVM: support vector machine; KNN: K-NearestNeighbor; RF: random decision forests
